# Supplementary material for: Lack of p38 activation in T cells increases IL-35 and protects against obesity by promoting thermogenesis
Source: EMBO Rep. 2024 May 10;25(6):11. doi: 10.1038/s44319-024-00149-y (PMC11169359; doi:10.1038/s44319-024-00149-y)
Supplement: Supplementary file 1 — Appendix [file 44319_2024_149_MOESM1_ESM.pdf]

Appendix

Appendix Figure S1. p38 MAPK activation deficiency does not induce problems in T cell development and activation 1

Appendix Figure S2. Metabolic phenotyping of CD4-cre and MKK3/6^{CD4-KO} mice fed HFD 3

Appendix Figure S3. Mice lacking MKK3/6 in T cells tend to have higher BAT temperature after NE stimulation 4

Appendix Figure S4. Gating strategy for analysis of SVF isolated from AT from HFD-fed mice ... 5

Appendix Figure S5. MKK3/6 deficiency in T cells promotes Treg cell accumulation in iWAT and BAT 6

Appendix Figure S6. MKK3/6 deficiency in T cells results increased Treg cells in lymph nodes, blood and eWAT after 2 weeks of HFD 7

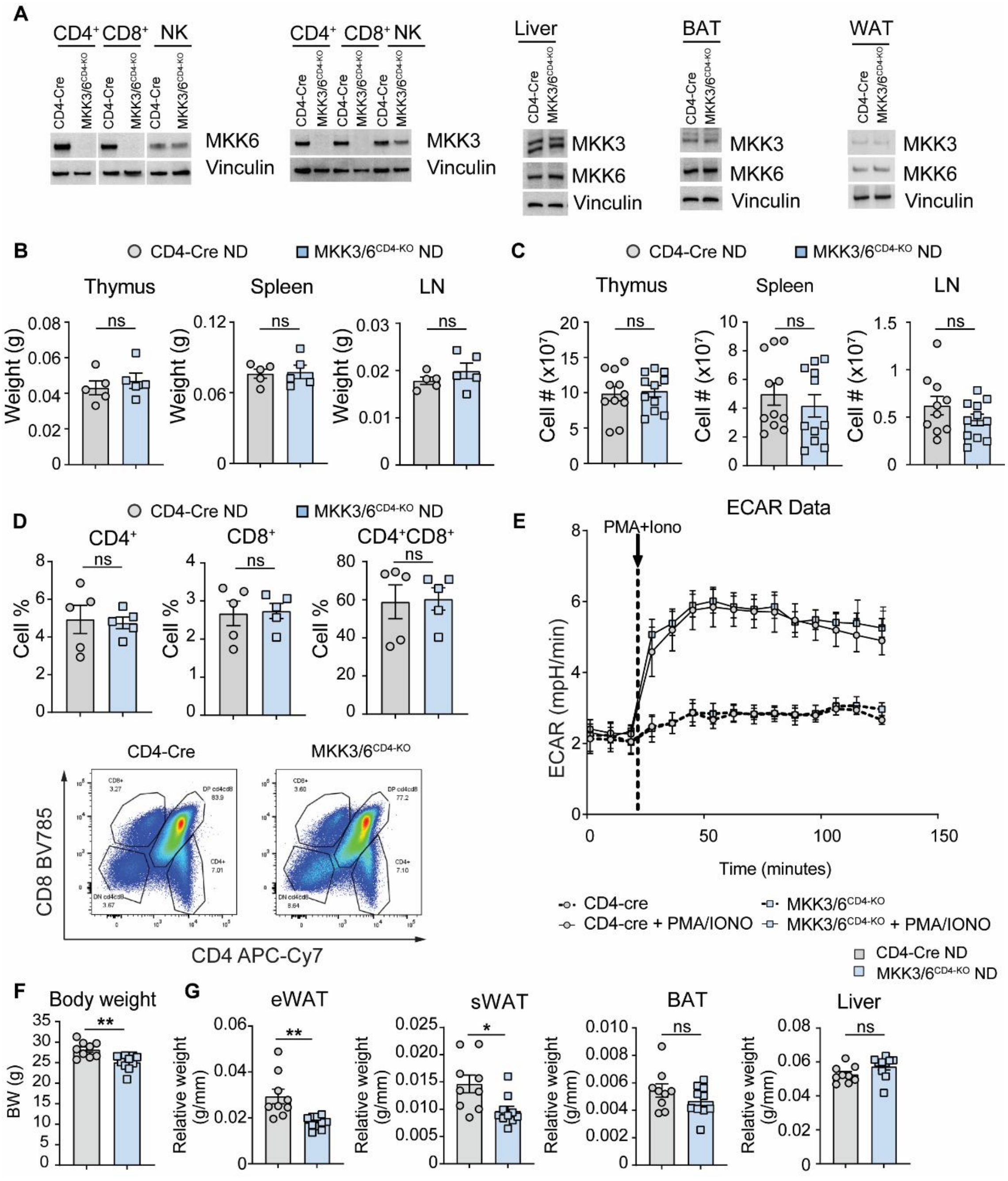

Appendix Figure S1. p38 MAPK activation deficiency does not induce problems in T cell development and activation.

(A) Western blot analysis of MKK3 and MKK6 expression in $CD4^+$ T cells, $CD8^+$ T cells, NK cells, liver, brown adipose tissue (BAT), and white adipose tissue (WAT) isolated from MKK3/6$^{CD4\text{-}KO}$ and control CD4-Cre mice. $CD4^+$ T cells, $CD8^+$ T cells and NK cells were sorted from spleen by FACS. **(B)** Thymus, spleen and periphery lymph nodes (LN) weight of 8-weeks old MKK3/6$^{CD4\text{-}KO}$ and control CD4-Cre mice fed

a normal diet (ND). (**C)** Cell number in thymus, spleen and LN of 8-weeks old control CD4-Cre and MKK3/6^{CD4-KO} mice. **(D)** Frequency of CD4$^+$, CD8$^+$ and CD4$^+$CD8$^+$ T cells in thymus and representative dot plots of FACS analysis of 8-weeks old control CD4-Cre and MKK3/6^{CD4-KO} mice. **(E)** Seahorse analysis of real time activation of naïve CD4$^+$ T cells (100.000 cells/well) from CD4-Cre and MKK3/6^{CD4-KO} mice with PMA/Ionomycin (50 and 500 ng/ml respectively). (**F)** Body weight evolution in CD4-Cre and MKK3/6^{CD4-KO} male (8–10-wk-old) mice fed the ND for 10 weeks. Data are presented as the increase above initial weight (left panel), and total weight at 18 weeks of age (right panel). (**G)** eWAT, sWAT, BAT, and liver mass relative to tibia length. Data Information: Data are presented as mean ± SEM, *p < 0.05, ** p < 0.01, ns: not significant. Analysis by *t* test or Welch's test when variances were different **(B, C, D, F)**. n= 5-11 biologically independent mice **(B, C, D, F, G)** or n=5-6 biologically independent wells **(E)** for each group, represented as single dots in the graphs.

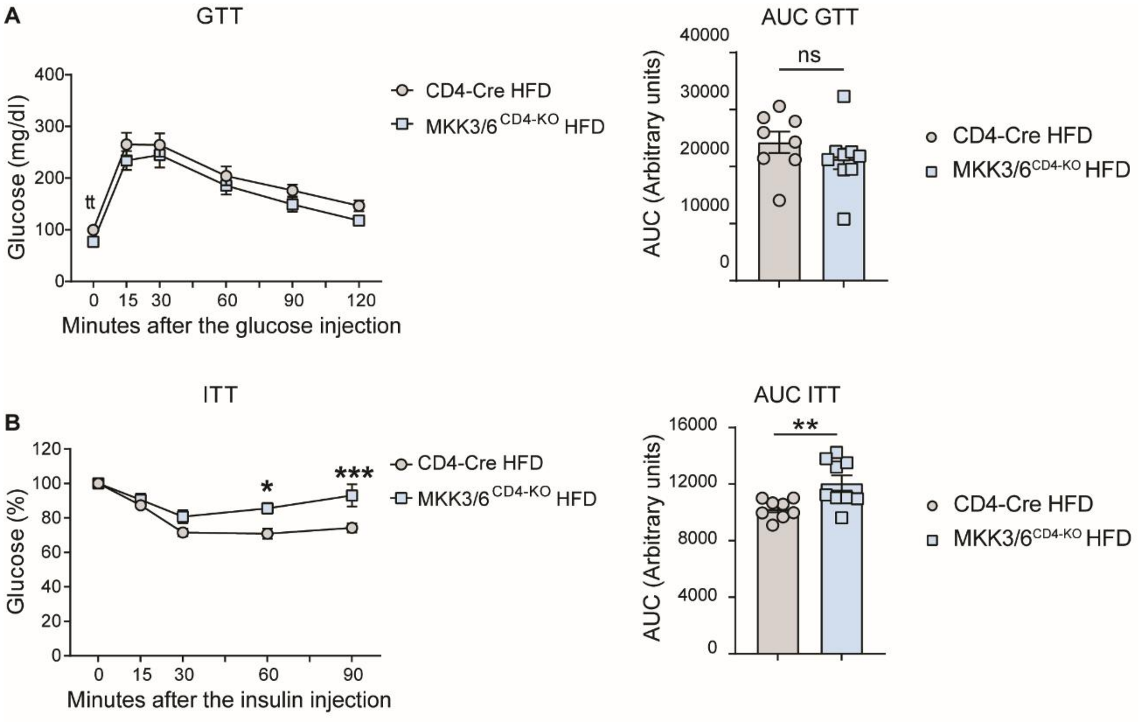

Appendix Figure S2. Metabolic phenotyping of CD4-cre and MKK3/6$^{CD4\text{-}KO}$ mice fed HFD.

(A-B) MKK3/6$^{CD4\text{-}KO}$ and control CD4-Cre mice were HFD-fed for 8 weeks. Mice were fasted overnight (for GTT) (**A**) or 1 hour (for ITT) (**B**), and blood glucose concentration was measured in mice given intraperitoneal injections of glucose (1 g/kg of total body weight) or insulin (0.75 U/kg of total body weight). Data Information: Data are presented as mean ± SEM, *p < 0.05, ** p < 0.01, ***p < 0.001, ns: not significant. Analysis by 2-way ANOVA **(A, B)**. n=8-9 biologically independent mice for each group, represented as single dots in the graphs.

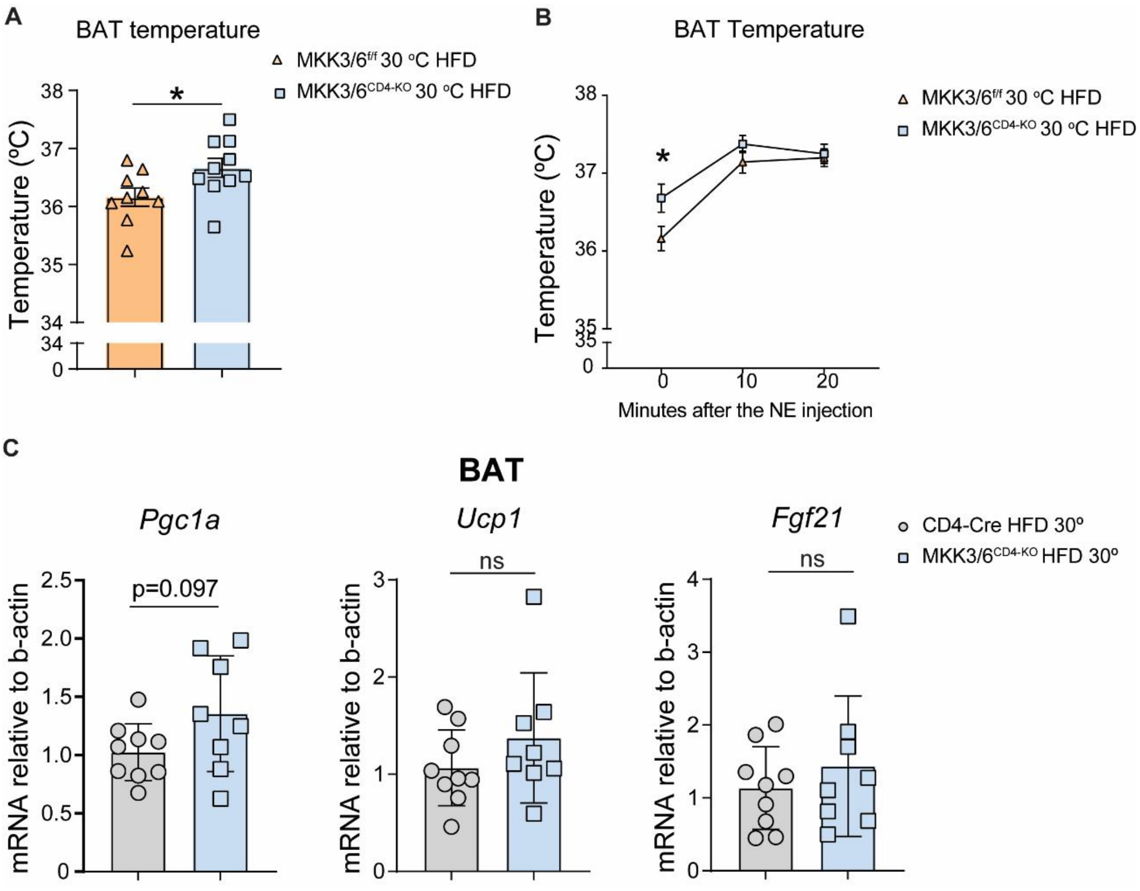

Appendix Figure S3. Mice lacking MKK3/6 in T cells tend to have higher BAT temperature after NE stimulation.

(A-B) MKK3/6$^{CD4\text{-}KO}$ and MKK3/6$^{f/f}$ mice were fed a high-fat diet (HFD) for 4 weeks and housed at thermoneutrality. Interscapular BAT temperature at basal conditions **(A)** and after NE injection (1 mg/kg of BW, i.p.) **(B)**. **(C)** qRT-PCR analysis of thermogenic gene mRNA expression in BAT isolated from CD4-Cre or MKK3/6$^{CD4\text{-}KO}$ mice. mRNA expression was normalized to the expression of β-actin mRNA. Data Information: Data are presented as mean ± SEM, *$p < 0.05$, ns: not significant. Exact p-values are shown. Analysis by *t* test **(A, C)** or 2-way ANOVA coupled to the Bonferroni post-test **(B)**.

n=8-10 biologically independent mice for each group, represented as single dots in the graphs.

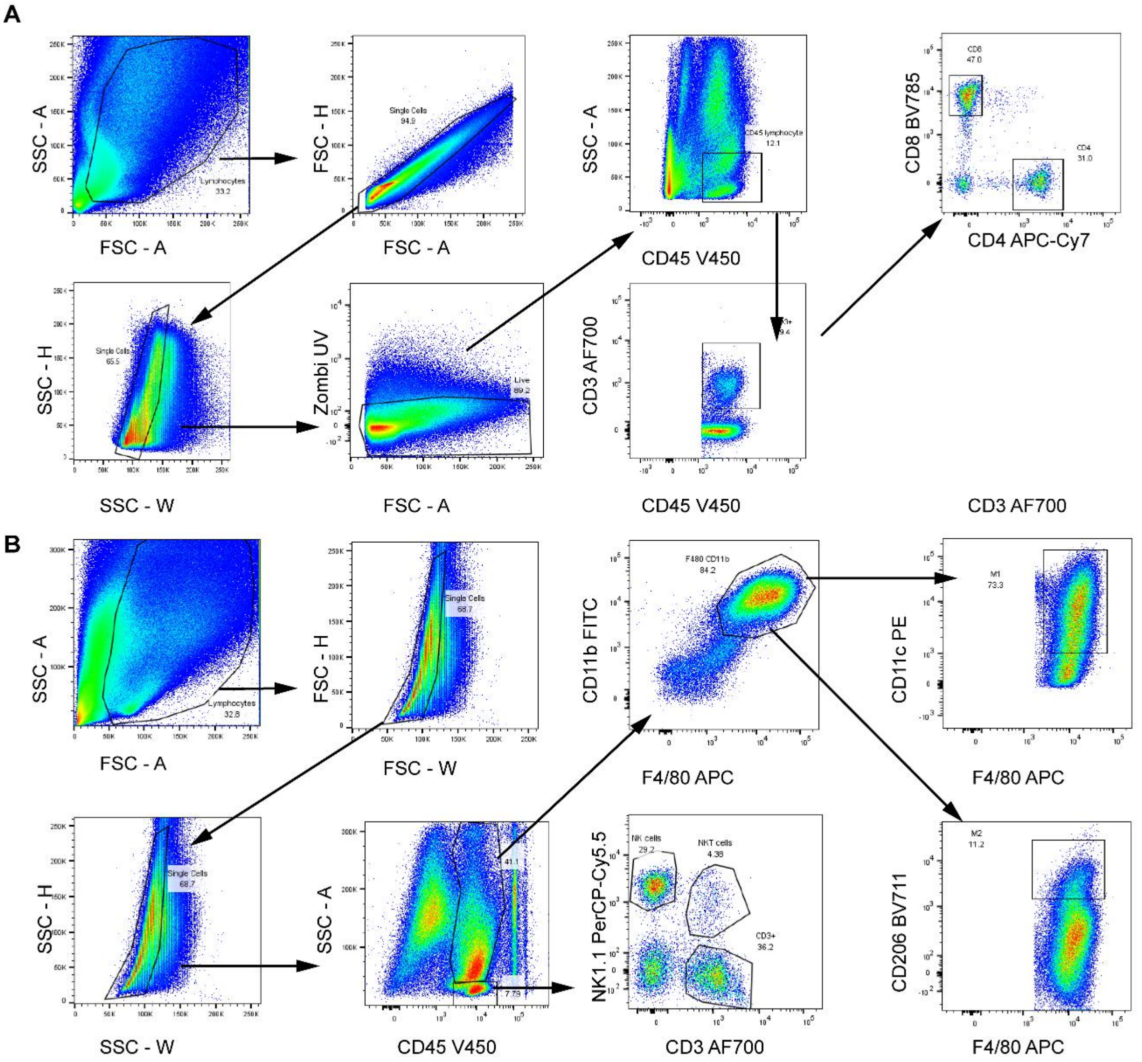

Appendix Figure S4. Gating strategy for analysis of SVF isolated from AT from HFD-fed mice.

(A-B) Representative dot plots illustrating the gating strategy for the analysis of lymphoid and myeloid populations: (**A**) $CD8^+$ cells ($Zombi^-CD45^+CD3^+CD8^+CD4^-$), $CD4^+$ cells ($Zombi^-CD45^+CD3^+CD4^+CD8^-$); (**B**) NK cells ($Dapi^-CD45^+NK1.1^+CD3^-$), M1 Mϕ ($Dapi^-CD45^+CD11b^+F4/80^+CD11c^+$), and M2 Mϕ ($Dapi^-CD45^+$ $CD11b^+F4/80^+CD206^+$). Mϕ, macrophage.

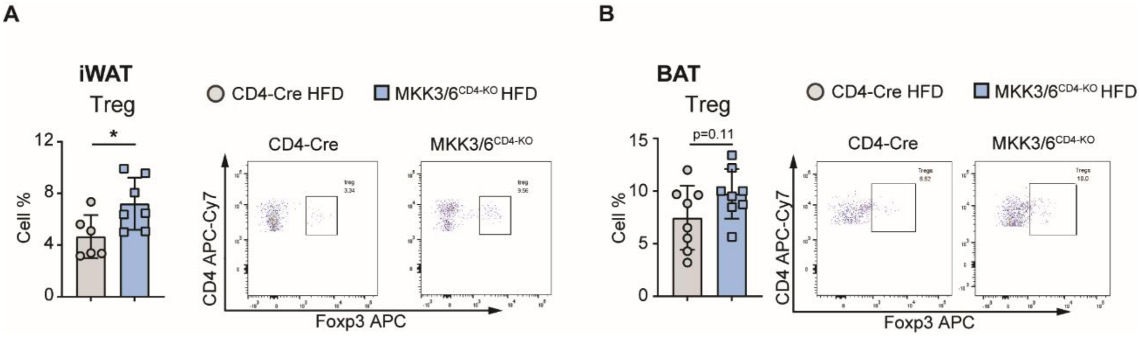

Appendix Figure S5. MKK3/6 deficiency in T cells promotes Treg cell accumulation in iWAT and BAT.

(A-B) FACS quantification and representative dot plots of Treg cells (CD4+ +Foxp3+) in inguinal WAT **(A)** and BAT **(B)**. Data information: Data are presented as mean ± SEM, $*p < 0.05$. Exact p-values are shown. Analysis by t test. n=6-8 biologically independent mice for each group, represented as single dots in the graphs.

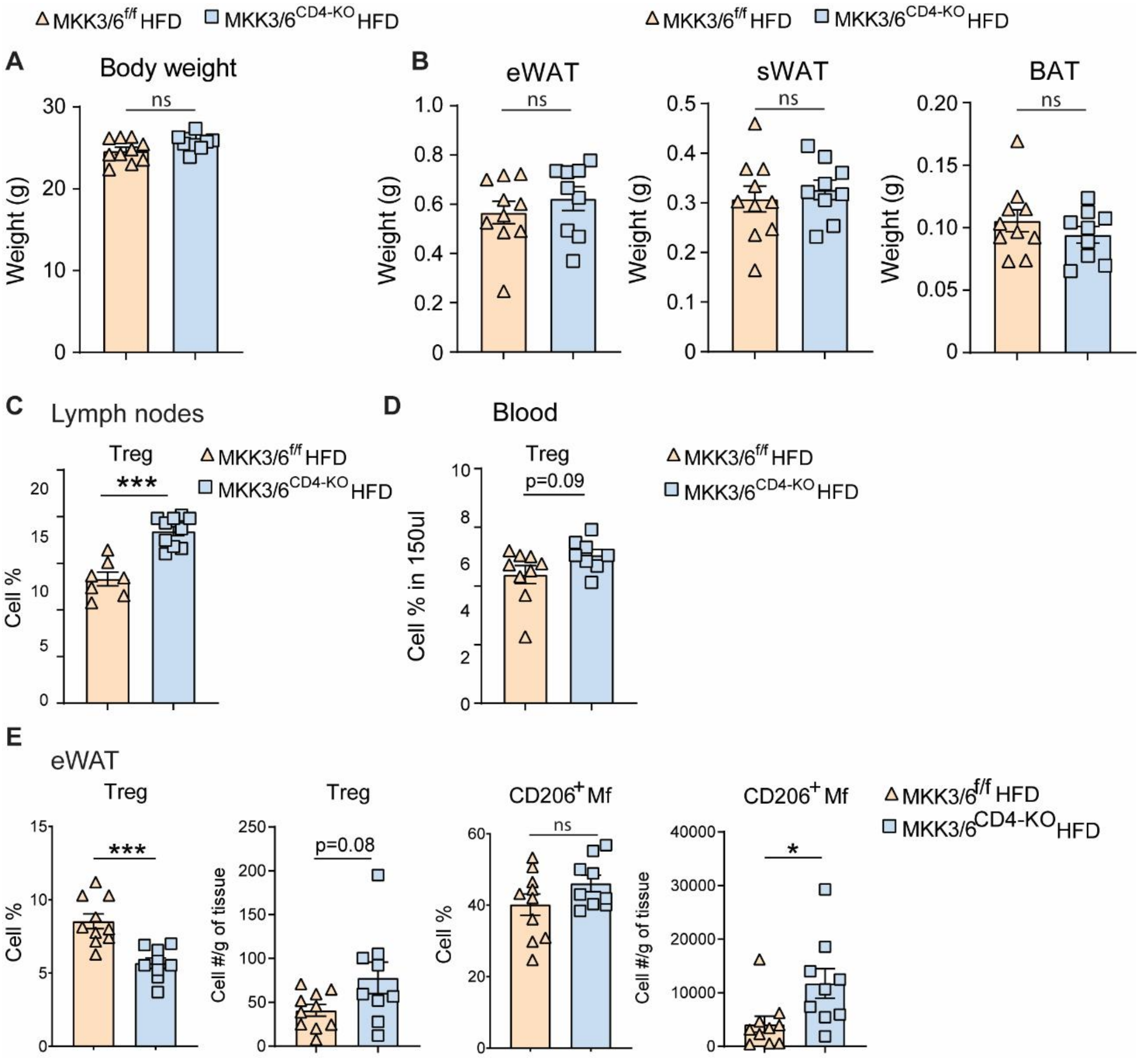

Appendix Figure S6. MKK3/6 deficiency in T cells results increased Treg cells in lymph nodes, blood and eWAT after 2 weeks of HFD.

(A-E) MKK3/6$^{CD4\text{-}KO}$ and MKK3/6$^{f/f}$ mice were fed with HFD for 2 weeks. Body **(A)** and fat depots **(B)** weight after 2 weeks of HFD. (**C, D)** FACS analysis of Treg cells in lymph nodes and blood. (**E)** FACS analysis of M2 macrophage population and Treg cells in eWAT tissue isolated from HFD-fed MKK3/6$^{CD4\text{-}KO}$ and control MKK3/6$^{f/f}$ mice. Data Information: Data are presented as mean ± SEM, *p < 0.05, ***p < 0.001, ns: not significant. Exact p values are shown. Analysis by *t* test (all graphs). n=9-10 biologically independent mice for each group, represented as single dots in the graphs.
