# Supplementary figures and images for: Lack of p38 activation in T cells increases IL-35 and protects against obesity by promoting thermogenesis
Source: EMBO Rep. 2024 May 10;25(6):11. doi: 10.1038/s44319-024-00149-y (PMC11169359; doi:10.1038/s44319-024-00149-y)

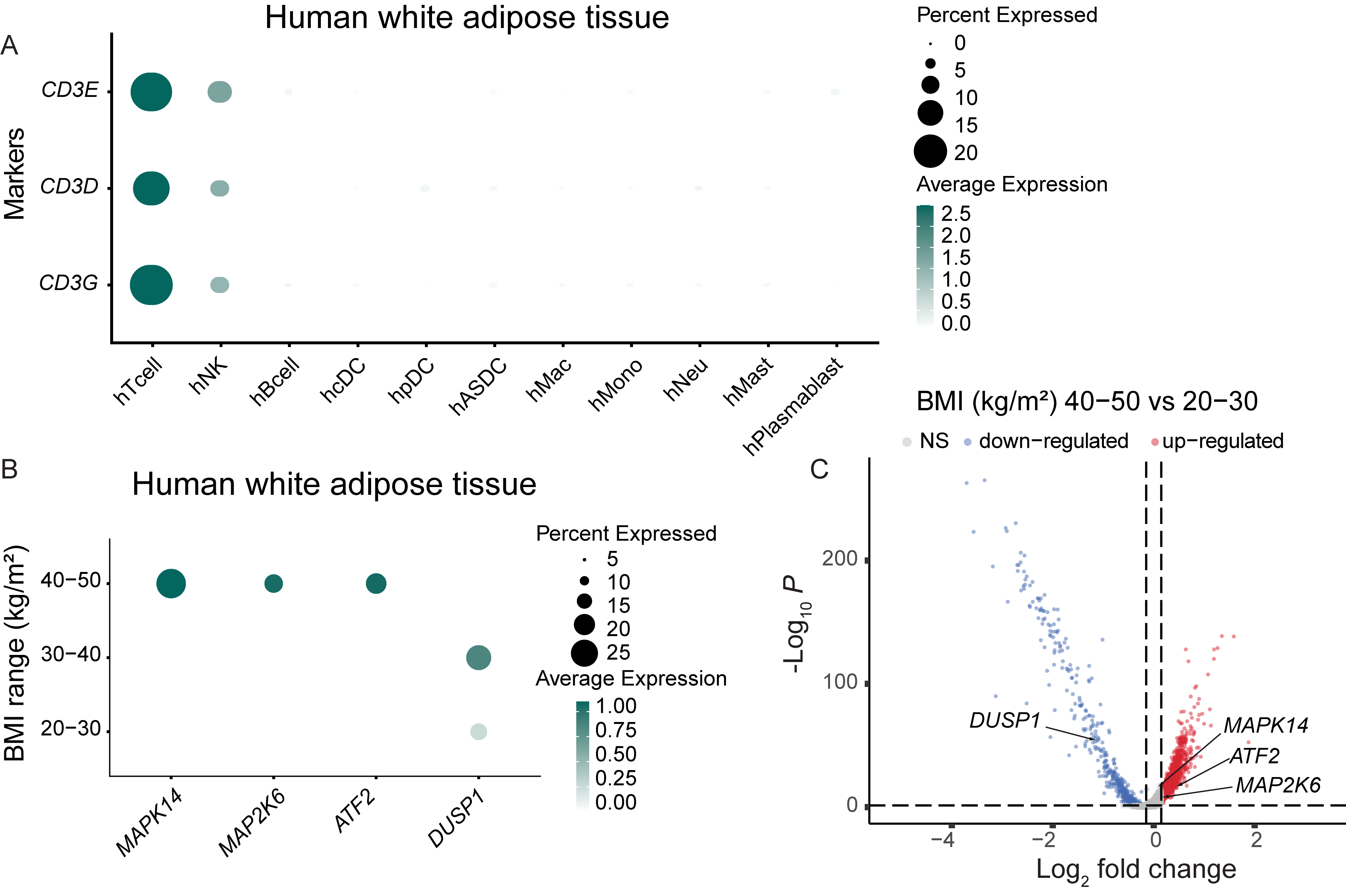

Supplement: Supplementary file 6 — Source data Fig. 1 [file 44319_2024_149_MOESM6_ESM.zip › Figure 1/Figure 1.tif]

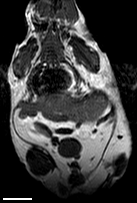

Supplement: Supplementary file 7 — Source data Fig. 2 [file 44319_2024_149_MOESM7_ESM.zip › Figure 2/2B/CD4-Cre with scale bar.tif]

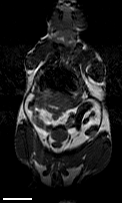

Supplement: Supplementary file 7 — Source data Fig. 2 [file 44319_2024_149_MOESM7_ESM.zip › Figure 2/2B/MKK3-6-CD4-KO with scale bar.tif]

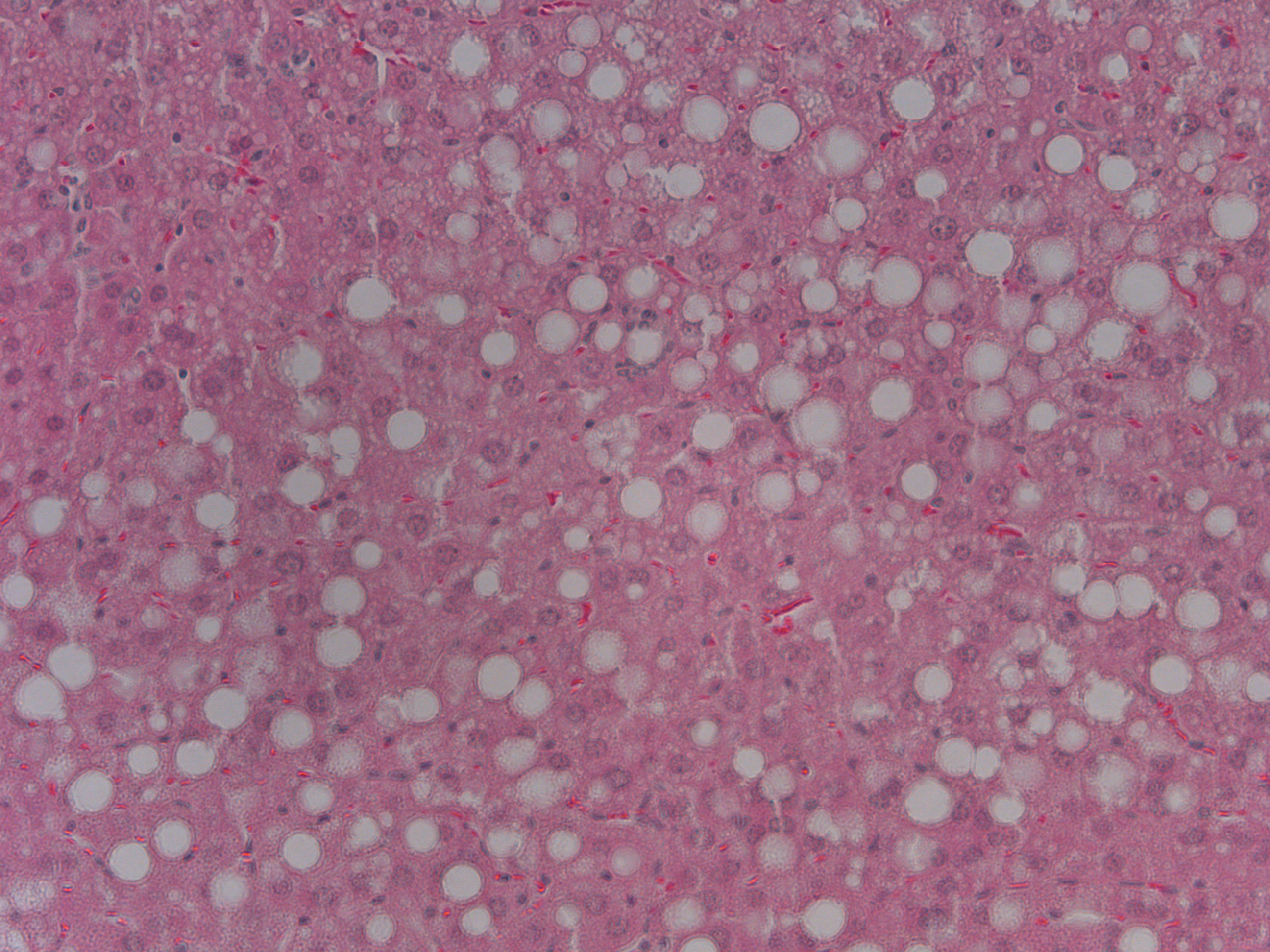

Supplement: Supplementary file 7 — Source data Fig. 2 [file 44319_2024_149_MOESM7_ESM.zip › Figure 2/2J/H&E CD4-Cre.tif]

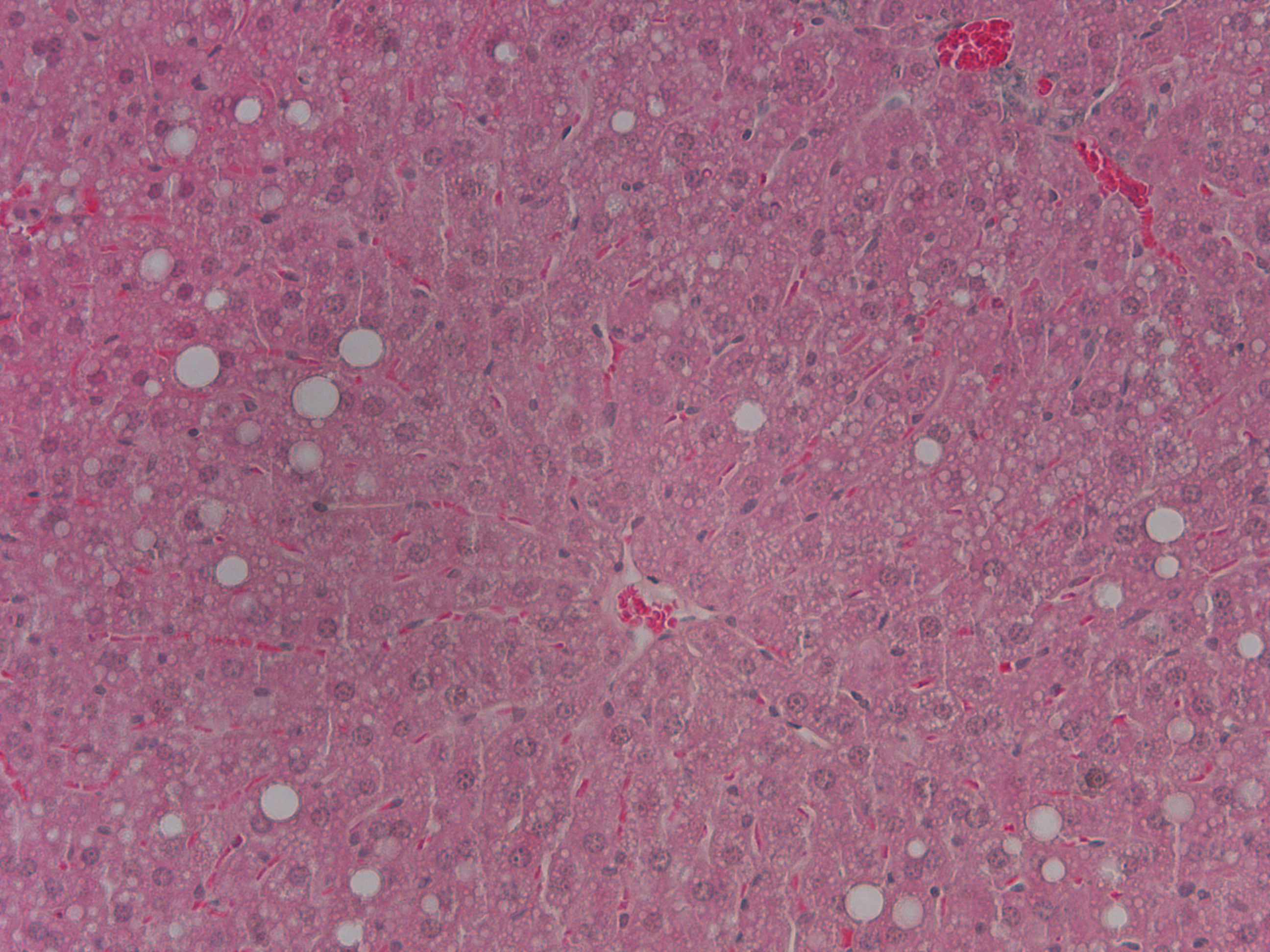

Supplement: Supplementary file 7 — Source data Fig. 2 [file 44319_2024_149_MOESM7_ESM.zip › Figure 2/2J/H&E MKK3-6-CD4-KO.tif]

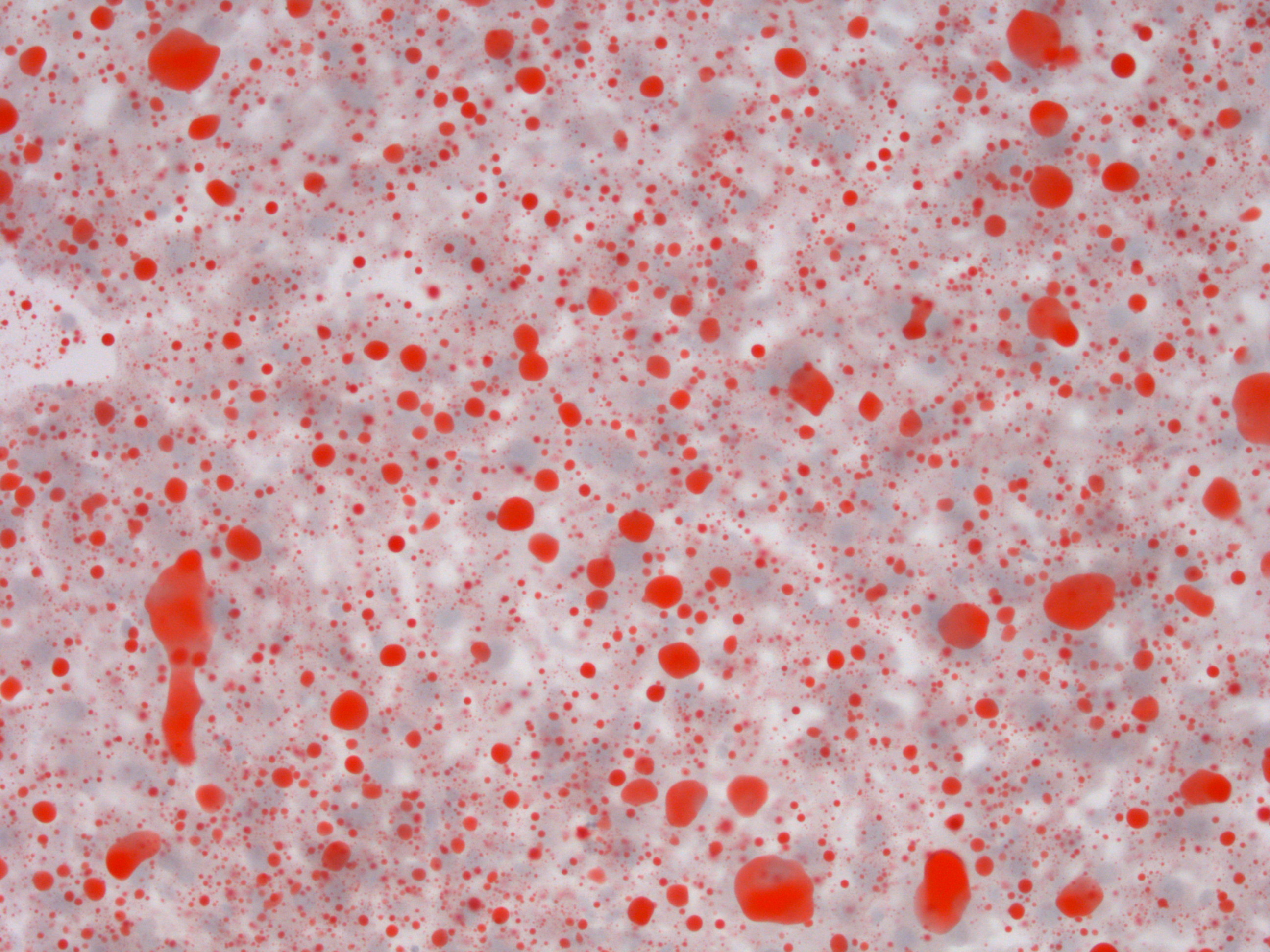

Supplement: Supplementary file 7 — Source data Fig. 2 [file 44319_2024_149_MOESM7_ESM.zip › Figure 2/2J/OilRed CD4-Cre.tif]

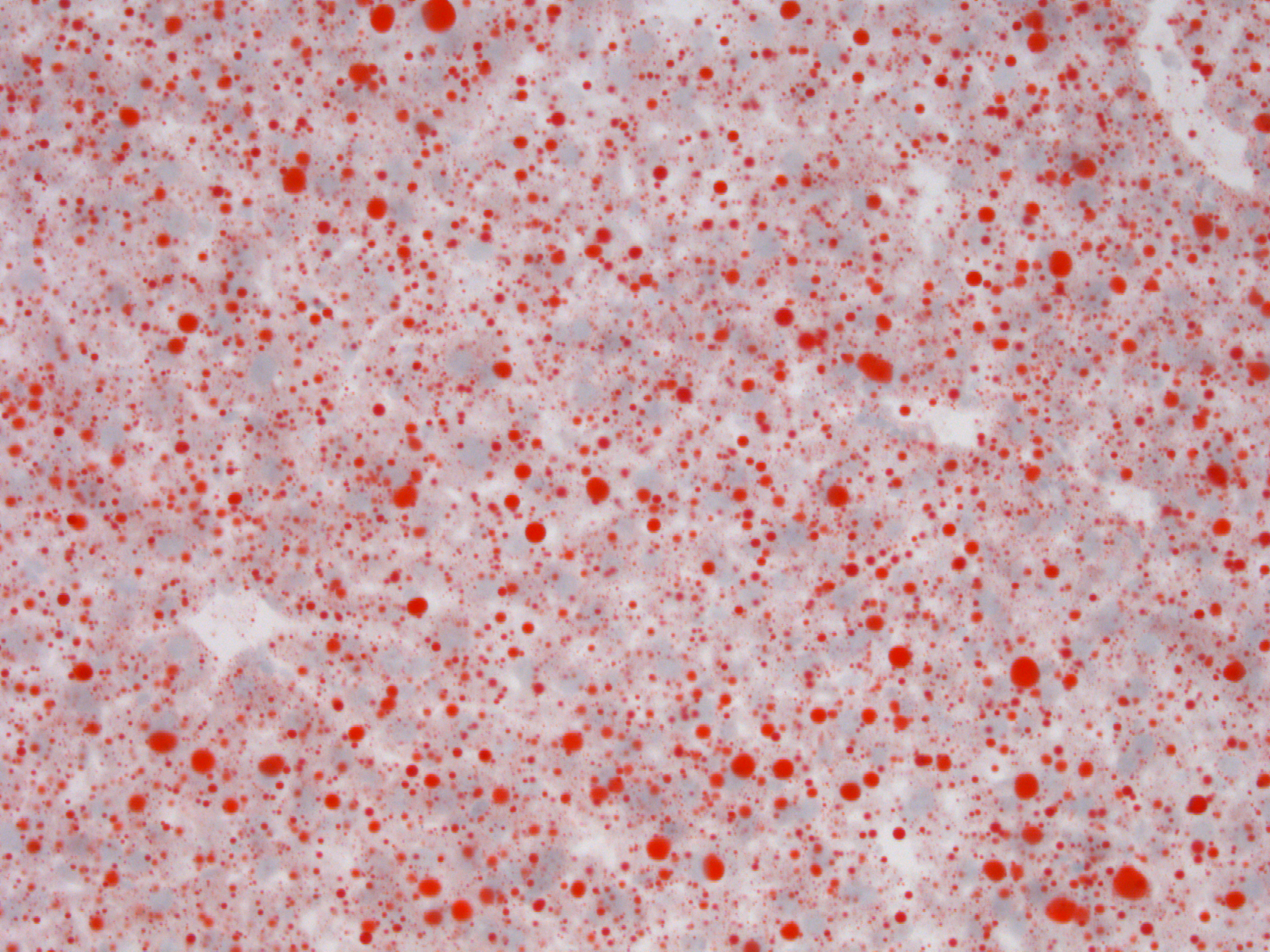

Supplement: Supplementary file 7 — Source data Fig. 2 [file 44319_2024_149_MOESM7_ESM.zip › Figure 2/2J/OilRed MKK3-6-CD4-KO.tif]

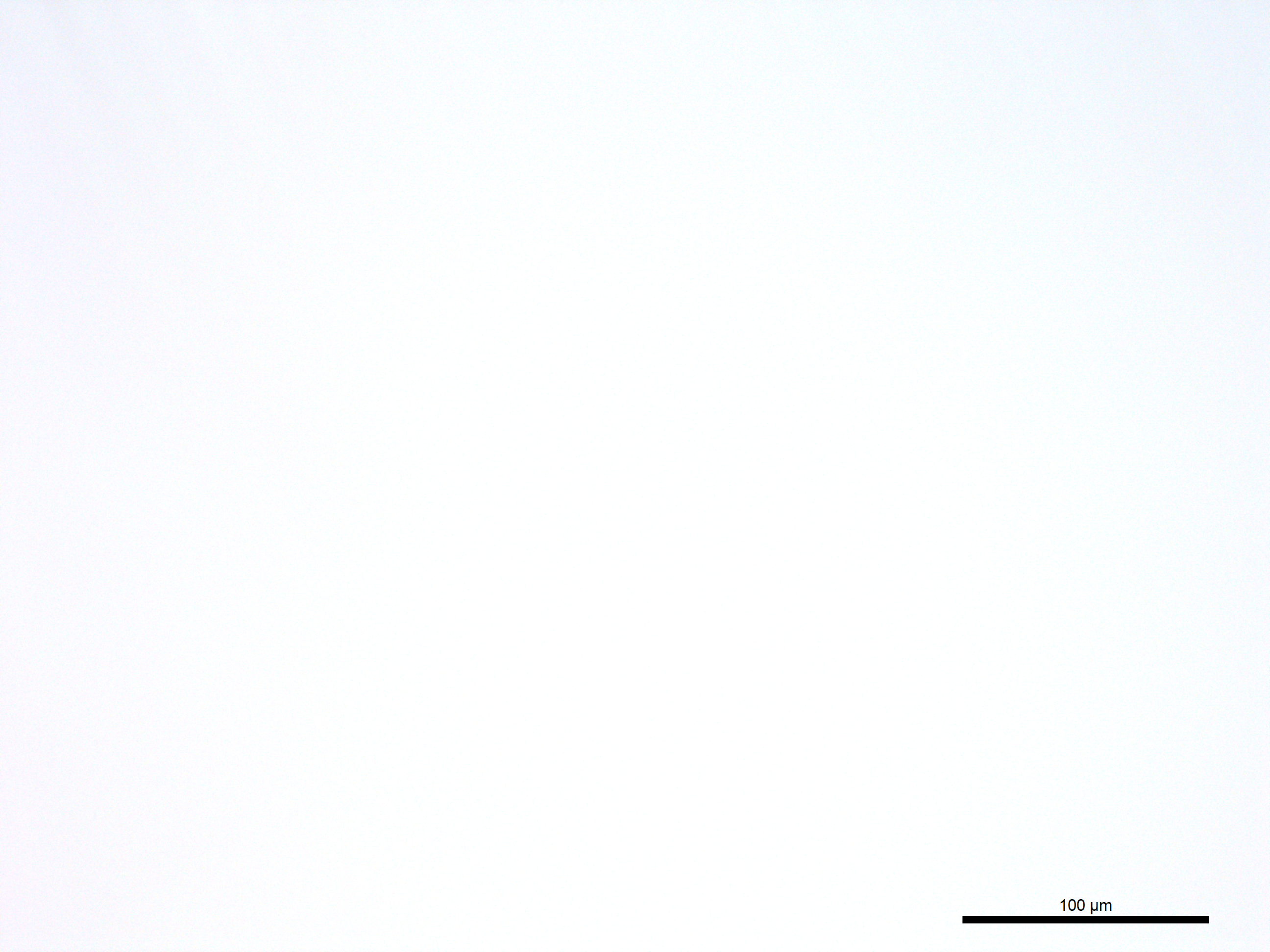

Supplement: Supplementary file 7 — Source data Fig. 2 [file 44319_2024_149_MOESM7_ESM.zip › Figure 2/2J/SCALE BAR.tif]

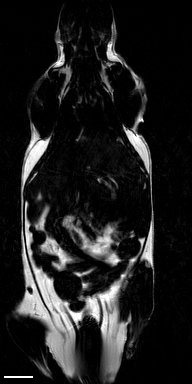

Supplement: Supplementary file 8 — Source data Fig. 3 [file 44319_2024_149_MOESM8_ESM.zip › Figure 3/3B/MKK36CD4KO with scale bar.jpg]

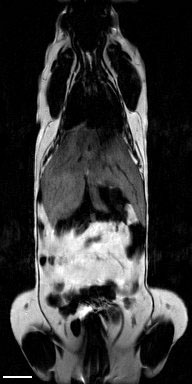

Supplement: Supplementary file 8 — Source data Fig. 3 [file 44319_2024_149_MOESM8_ESM.zip › Figure 3/3B/MKK36f_f with scale bar.jpg]

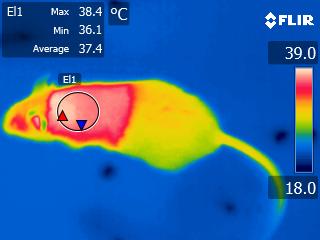

Supplement: Supplementary file 8 — Source data Fig. 3 [file 44319_2024_149_MOESM8_ESM.zip › Figure 3/3E/MKK36CD4KO.jpg]

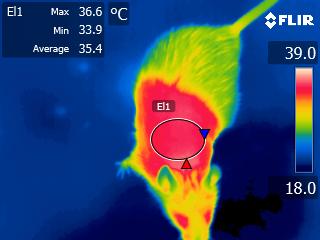

Supplement: Supplementary file 8 — Source data Fig. 3 [file 44319_2024_149_MOESM8_ESM.zip › Figure 3/3E/MKK36f_f.jpg]

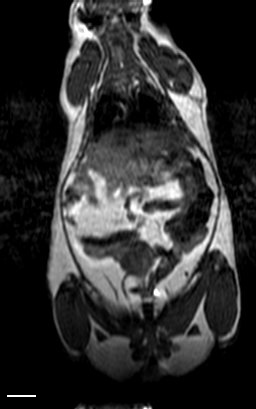

Supplement: Supplementary file 9 — Source data Fig. 4 [file 44319_2024_149_MOESM9_ESM.zip › Figure 4/4B/CD4-Cre with scale bar.jpg]

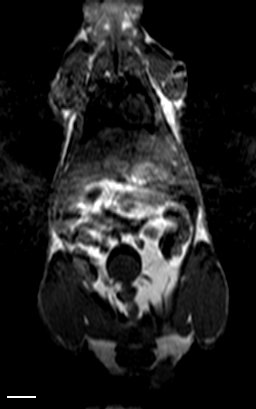

Supplement: Supplementary file 9 — Source data Fig. 4 [file 44319_2024_149_MOESM9_ESM.zip › Figure 4/4B/MKK3-6-CD4-KO with scale bar.jpg]

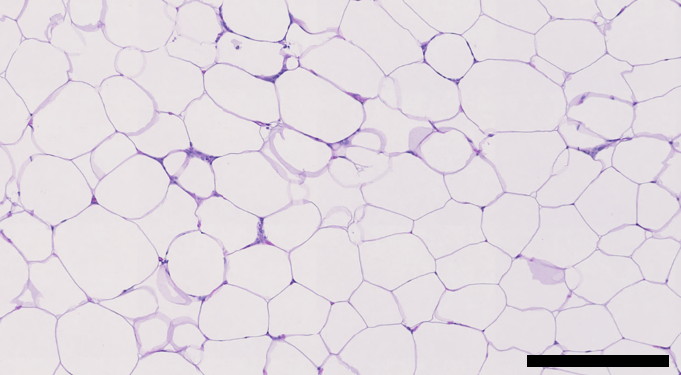

Supplement: Supplementary file 9 — Source data Fig. 4 [file 44319_2024_149_MOESM9_ESM.zip › Figure 4/4D/eWAT CD4-Cre.tif]

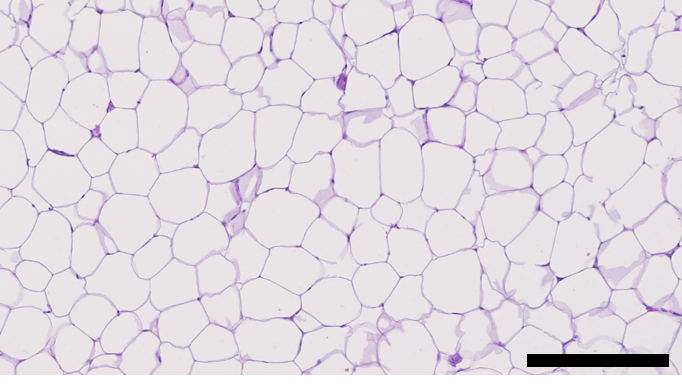

Supplement: Supplementary file 9 — Source data Fig. 4 [file 44319_2024_149_MOESM9_ESM.zip › Figure 4/4D/eWAT MKK36 KO.tif]

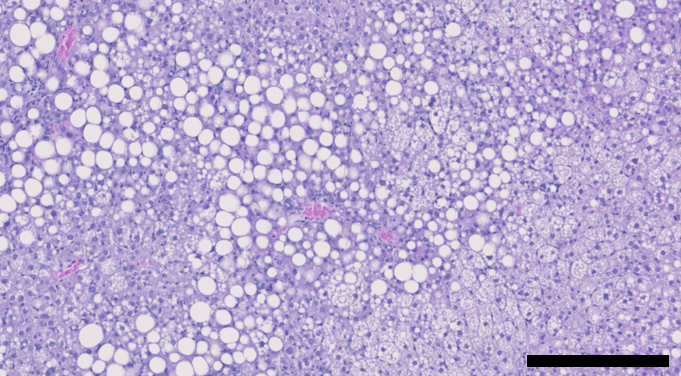

Supplement: Supplementary file 9 — Source data Fig. 4 [file 44319_2024_149_MOESM9_ESM.zip › Figure 4/4D/Liver CD4-Cre.tif]

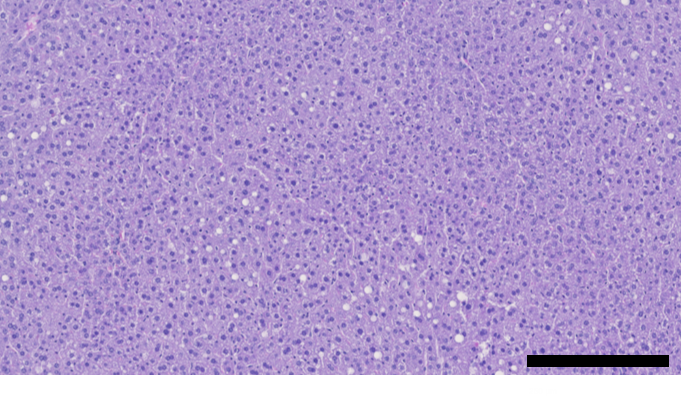

Supplement: Supplementary file 9 — Source data Fig. 4 [file 44319_2024_149_MOESM9_ESM.zip › Figure 4/4D/Liver MKK36 KO.tif]

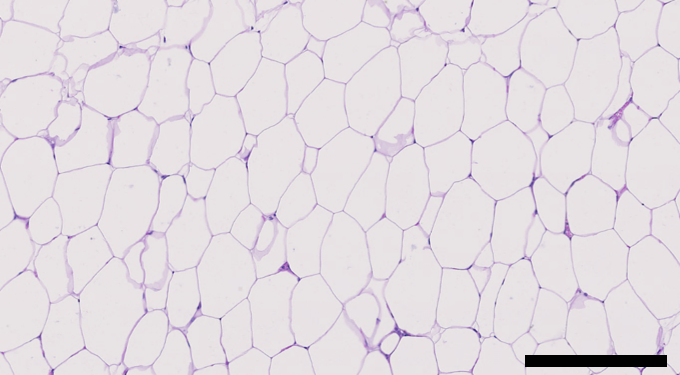

Supplement: Supplementary file 9 — Source data Fig. 4 [file 44319_2024_149_MOESM9_ESM.zip › Figure 4/4D/sWAT CD4-Cre.tif]

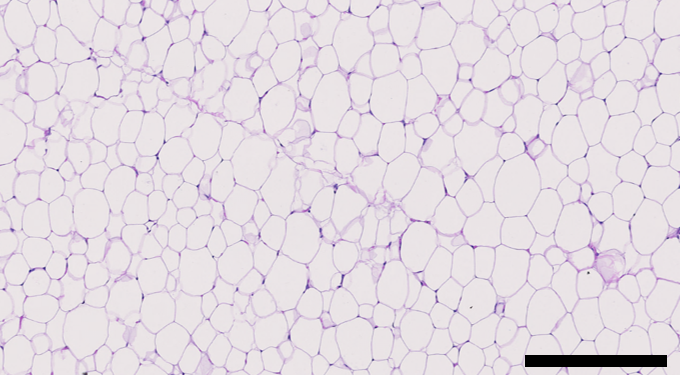

Supplement: Supplementary file 9 — Source data Fig. 4 [file 44319_2024_149_MOESM9_ESM.zip › Figure 4/4D/sWAT MKK36 KO.tif]

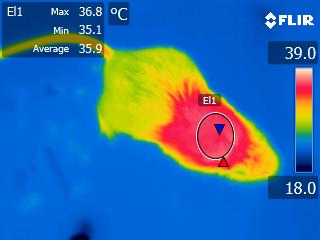

Supplement: Supplementary file 9 — Source data Fig. 4 [file 44319_2024_149_MOESM9_ESM.zip › Figure 4/4E/CD4-Cre.jpg]

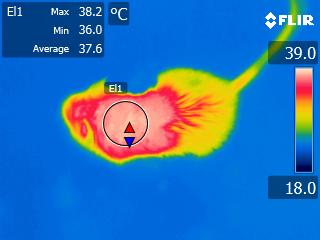

Supplement: Supplementary file 9 — Source data Fig. 4 [file 44319_2024_149_MOESM9_ESM.zip › Figure 4/4E/MKK3-6-CD4-KO.jpg]

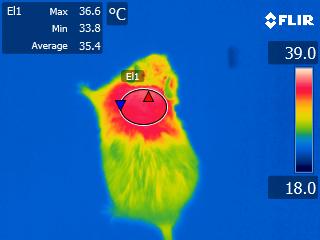

Supplement: Supplementary file 10 — Source data Fig. 5 [file 44319_2024_149_MOESM10_ESM.zip › Figure 5/5A/CD4-Cre.jpg]

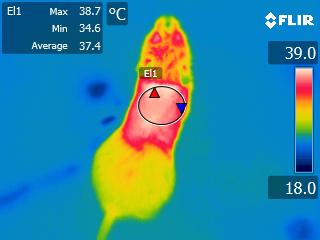

Supplement: Supplementary file 10 — Source data Fig. 5 [file 44319_2024_149_MOESM10_ESM.zip › Figure 5/5A/MKK3-6-CD4-KO.jpg]

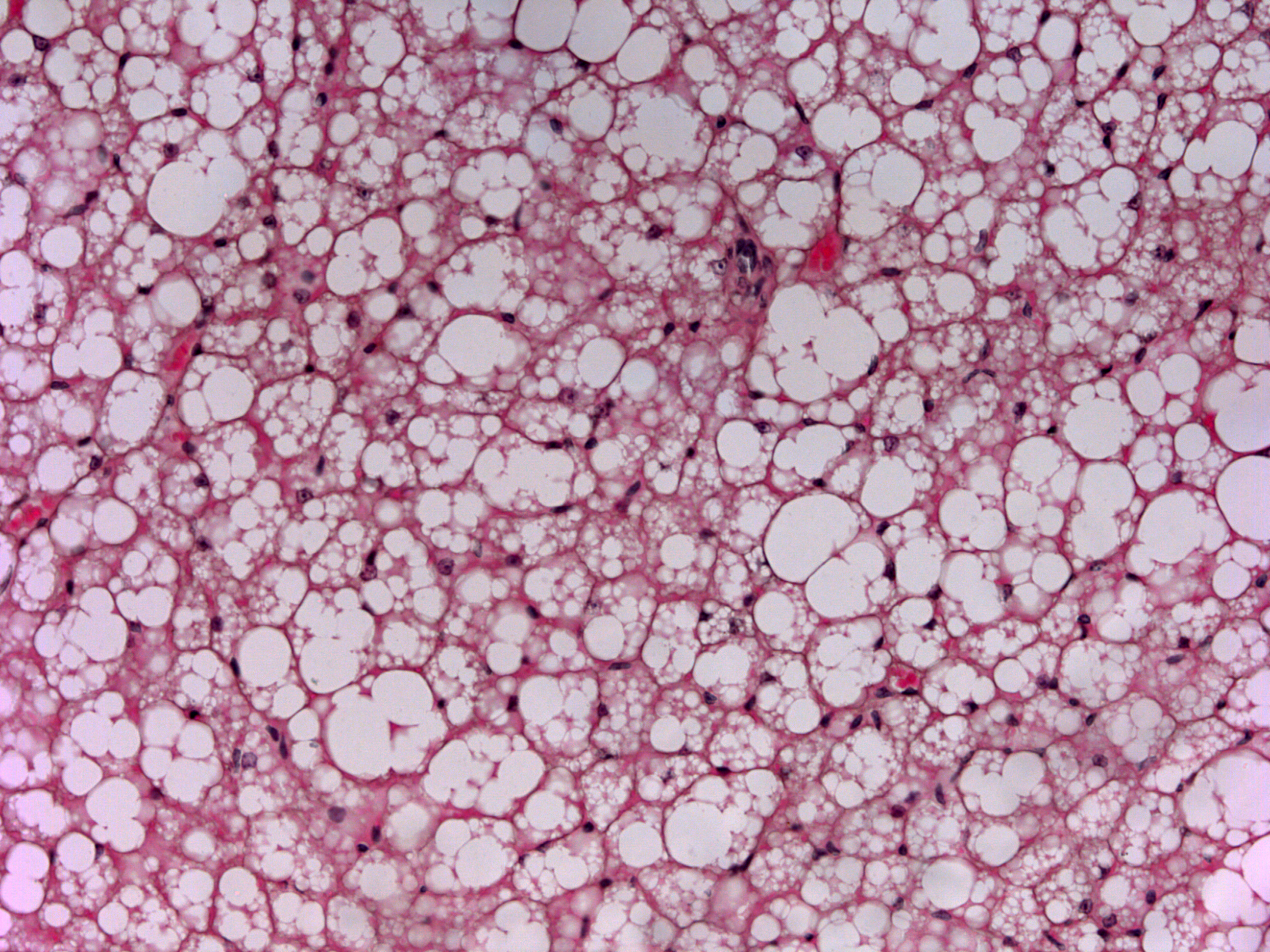

Supplement: Supplementary file 10 — Source data Fig. 5 [file 44319_2024_149_MOESM10_ESM.zip › Figure 5/5B/CD4-Cre.jpg]

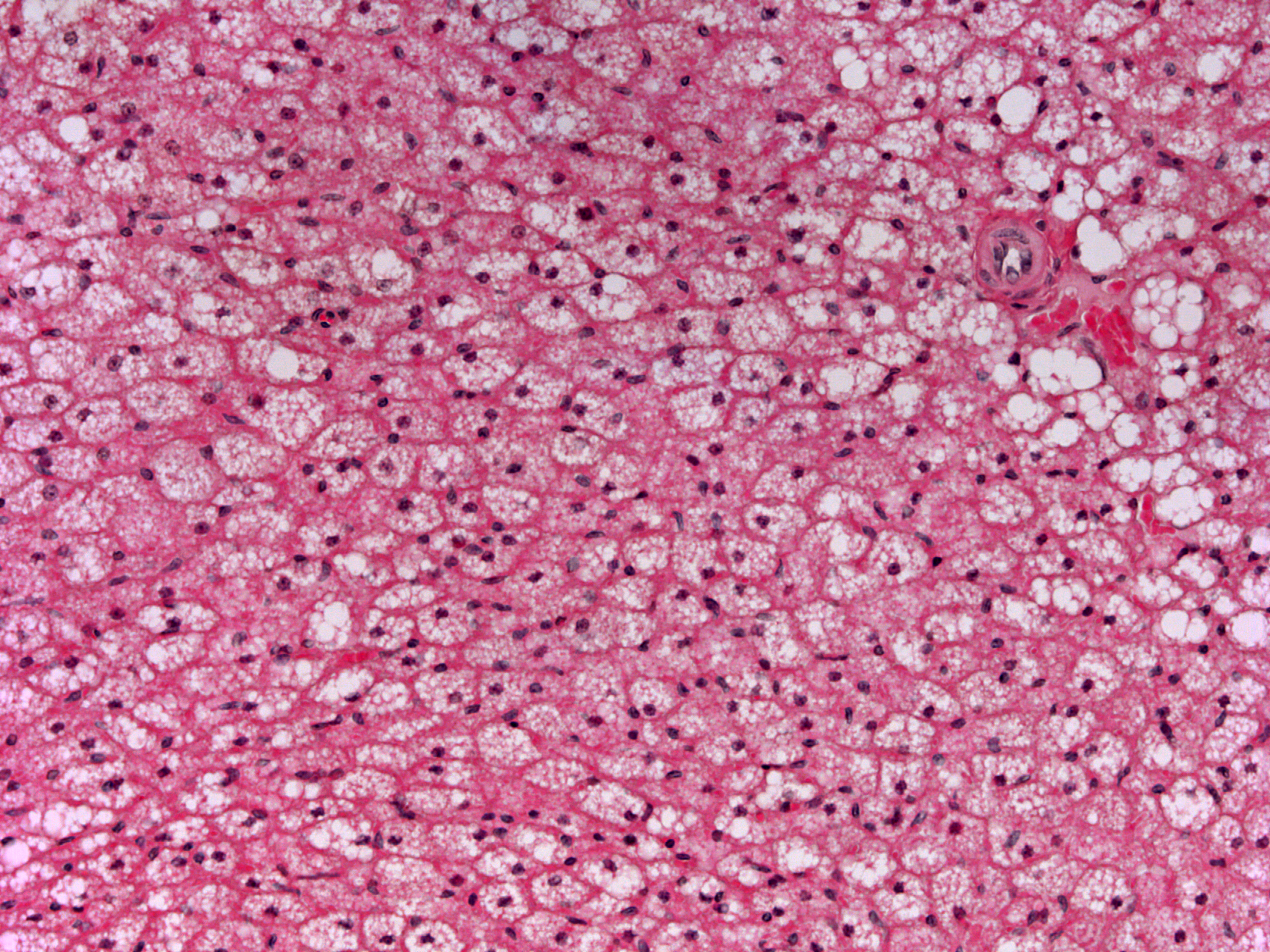

Supplement: Supplementary file 10 — Source data Fig. 5 [file 44319_2024_149_MOESM10_ESM.zip › Figure 5/5B/MKK3-6-CD4-KO.jpg]

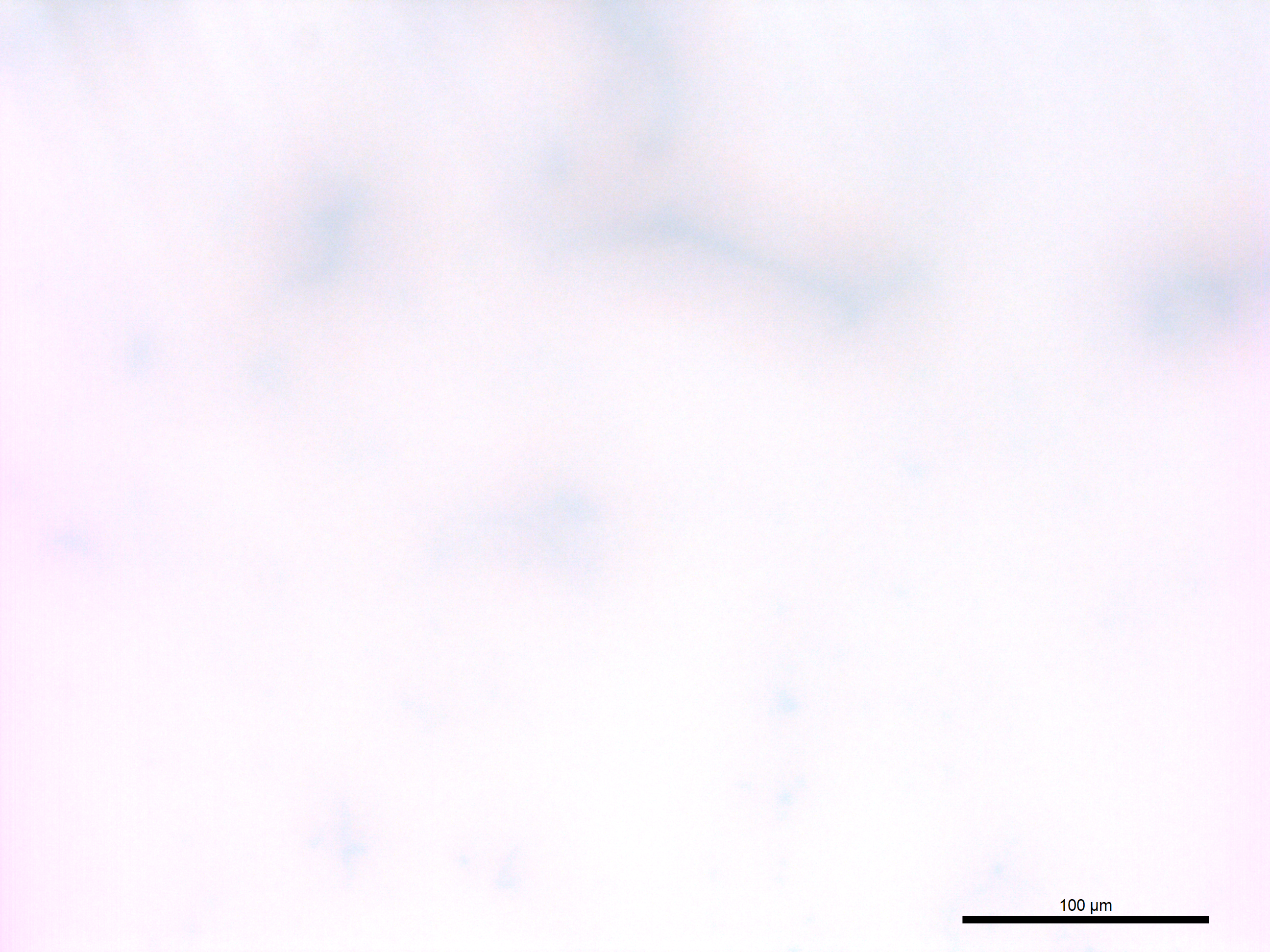

Supplement: Supplementary file 10 — Source data Fig. 5 [file 44319_2024_149_MOESM10_ESM.zip › Figure 5/5B/SCALE BAR 100 um.tif]

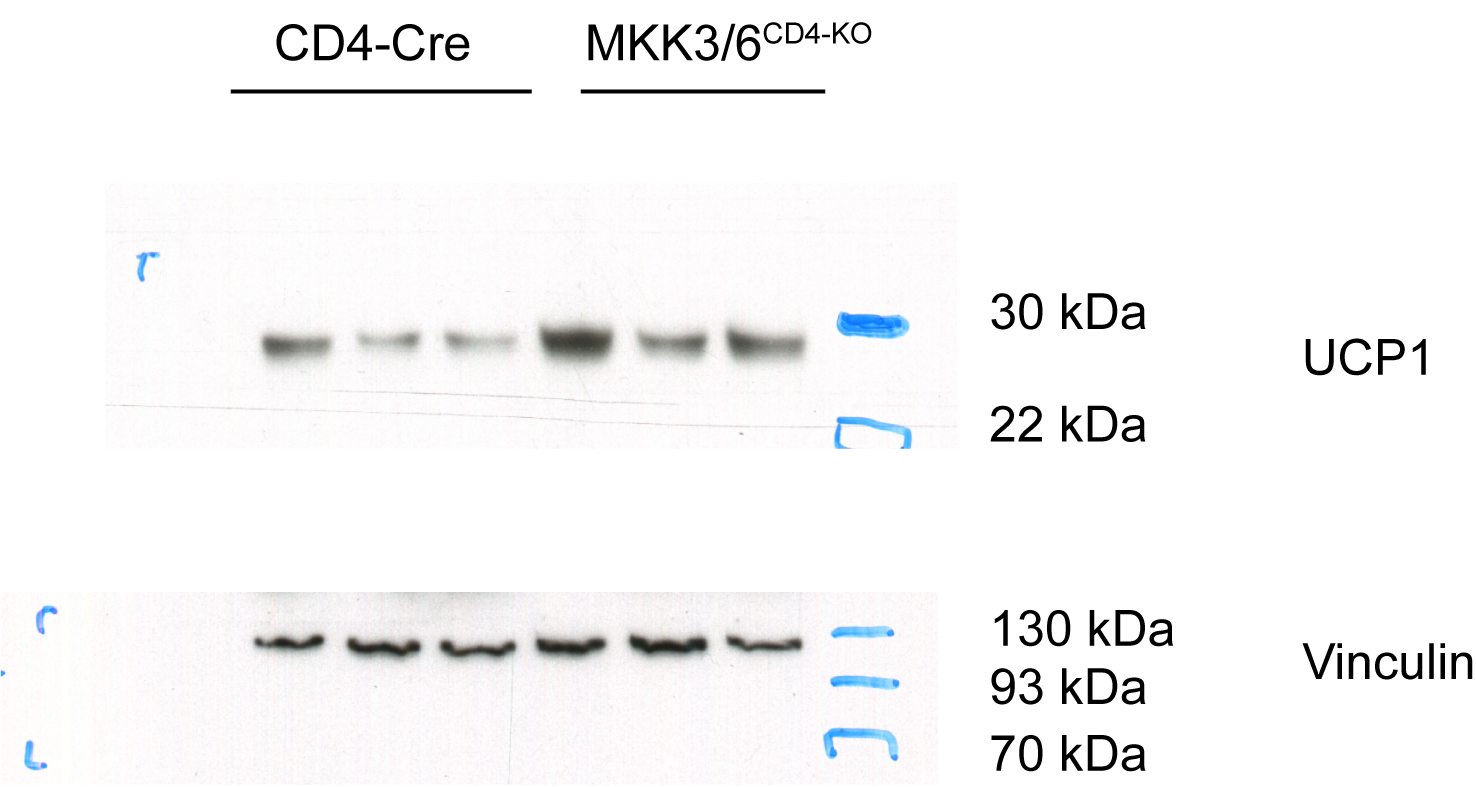

Supplement: Supplementary file 10 — Source data Fig. 5 [file 44319_2024_149_MOESM10_ESM.zip › Figure 5/5C/5C.tif]

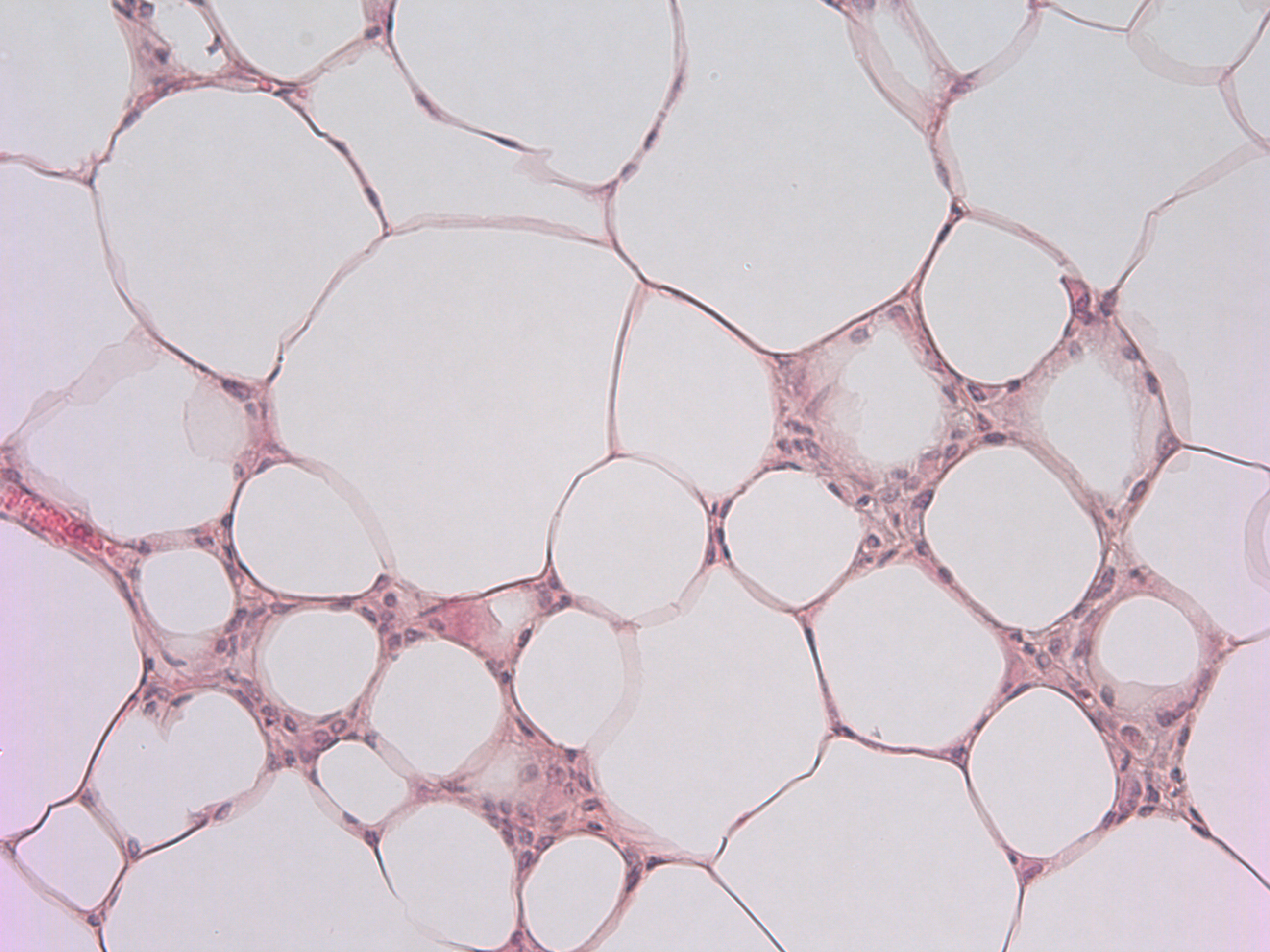

Supplement: Supplementary file 10 — Source data Fig. 5 [file 44319_2024_149_MOESM10_ESM.zip › Figure 5/5F/CD4-Cre.tif]

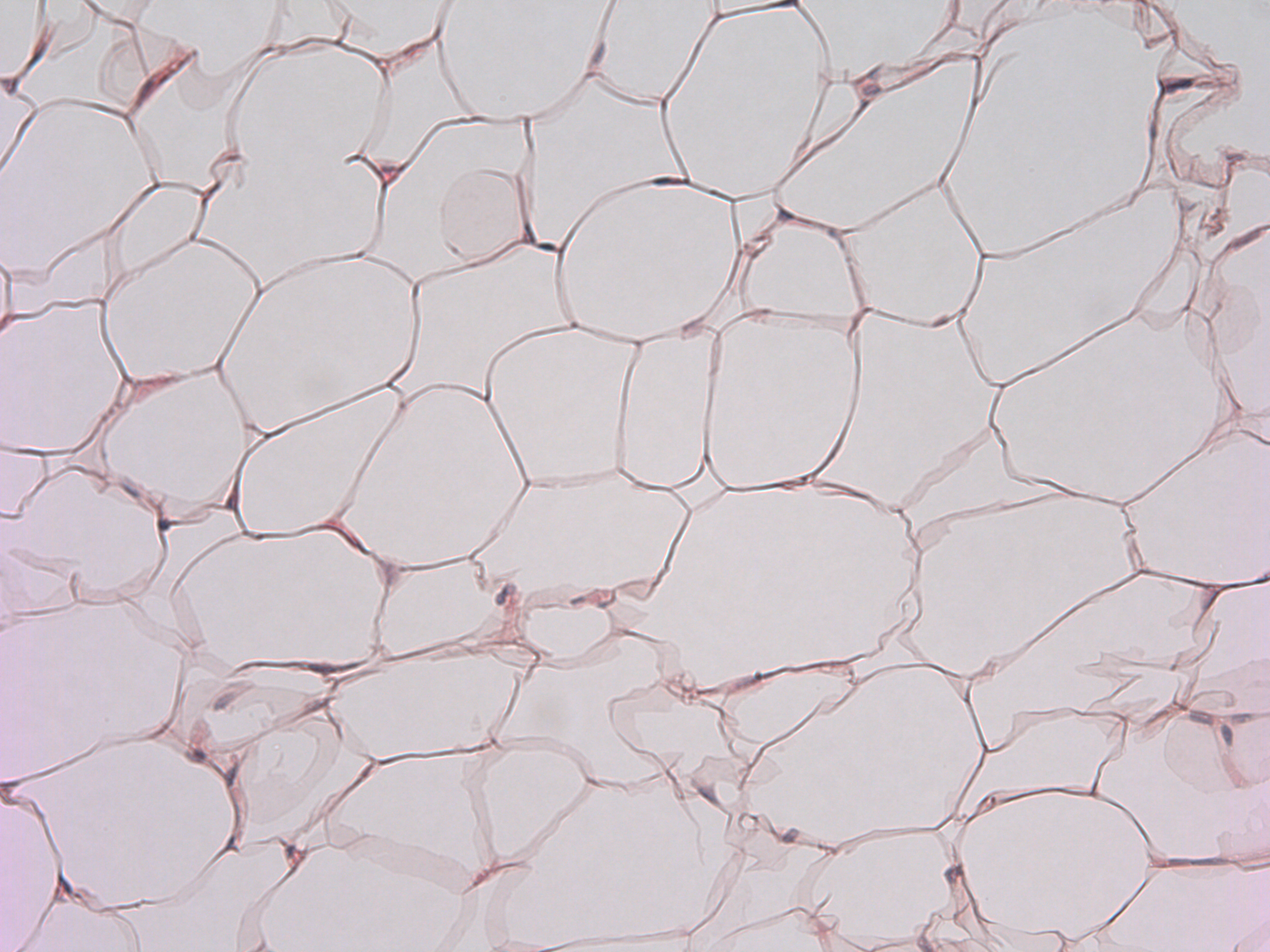

Supplement: Supplementary file 10 — Source data Fig. 5 [file 44319_2024_149_MOESM10_ESM.zip › Figure 5/5F/MKK3-6-CD4-KO.tif]

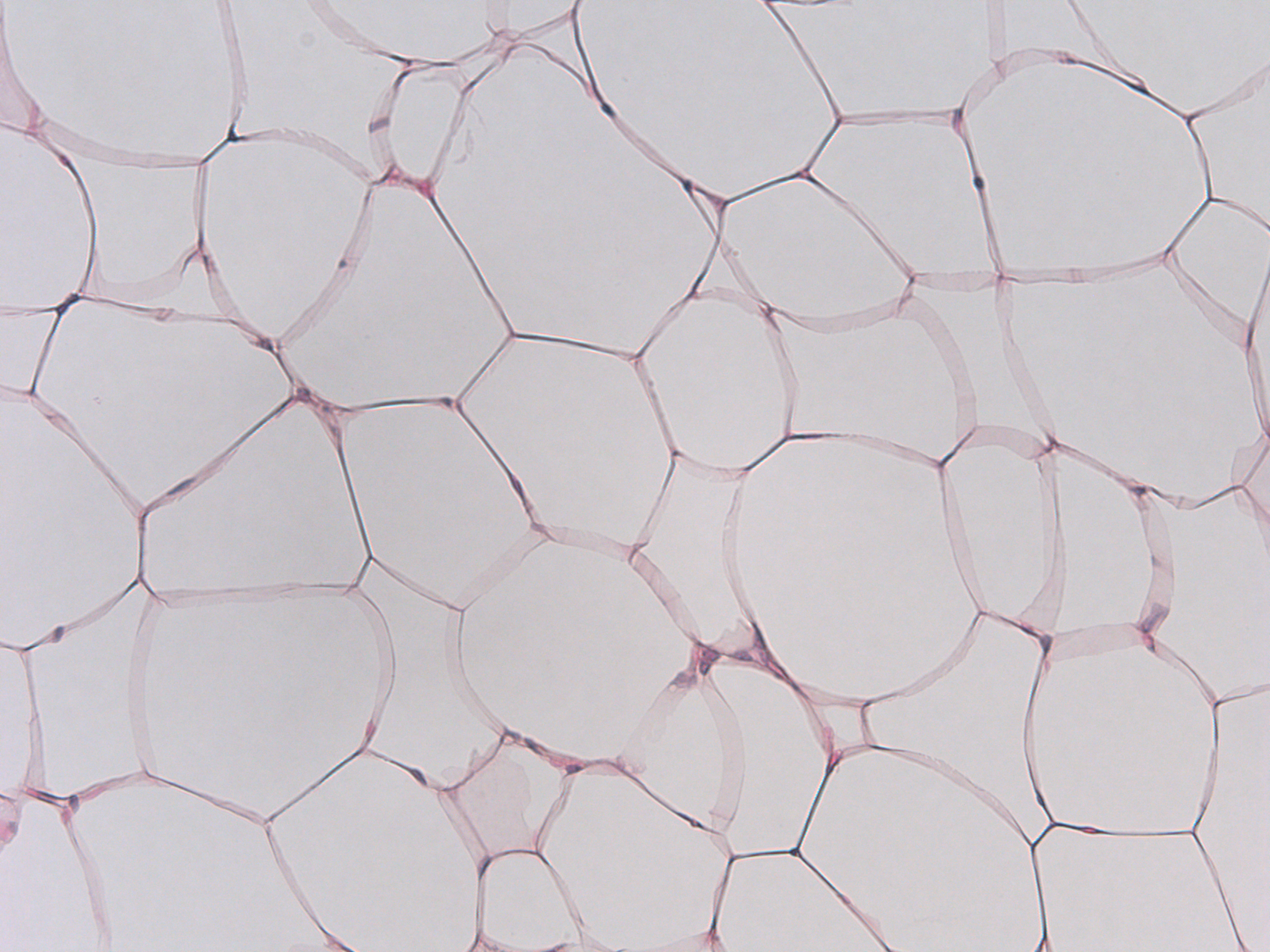

Supplement: Supplementary file 10 — Source data Fig. 5 [file 44319_2024_149_MOESM10_ESM.zip › Figure 5/5H/sWAT CD4CRE.tif]

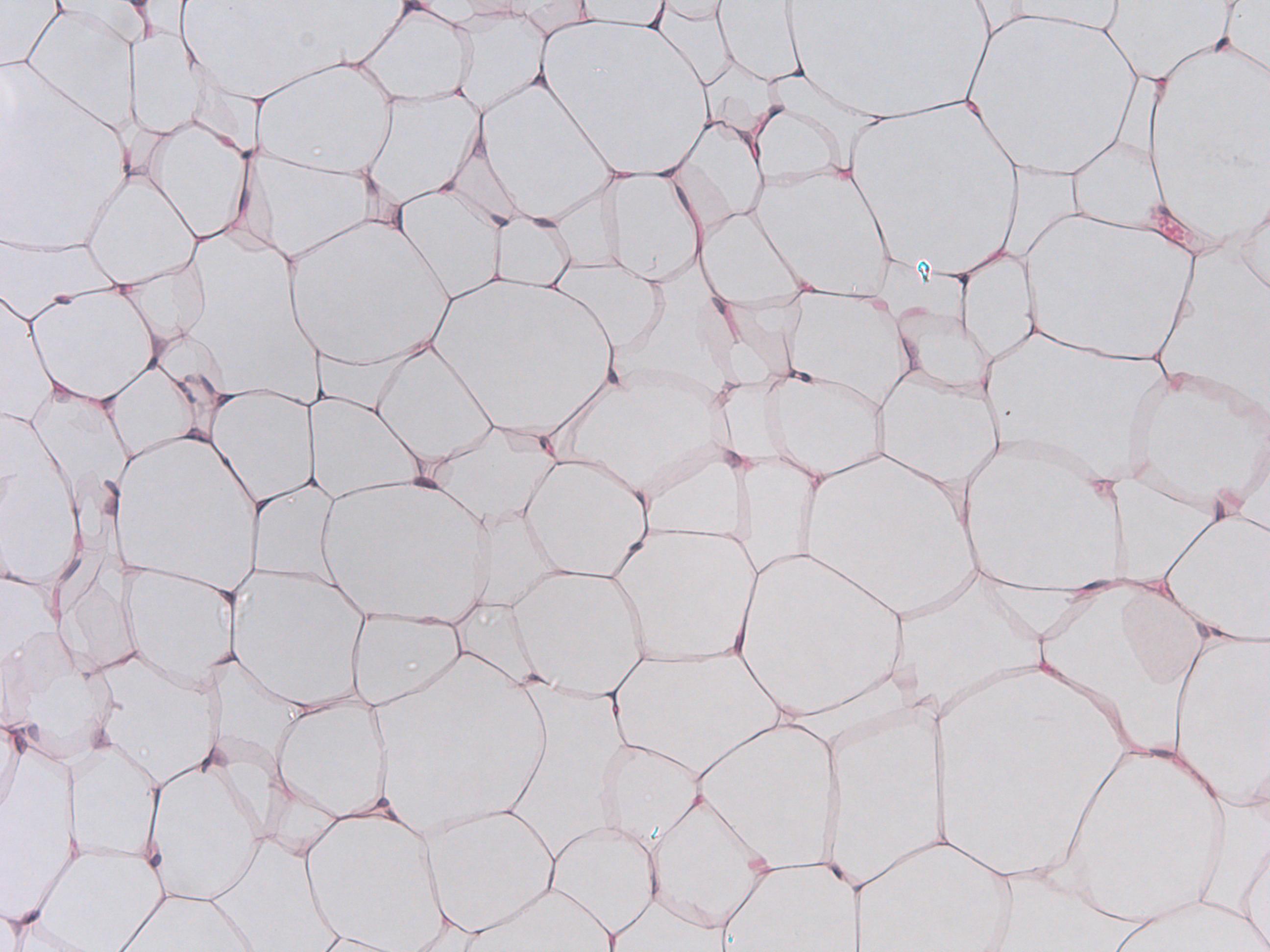

Supplement: Supplementary file 10 — Source data Fig. 5 [file 44319_2024_149_MOESM10_ESM.zip › Figure 5/5H/sWAT MKK36CD4KO.tif]

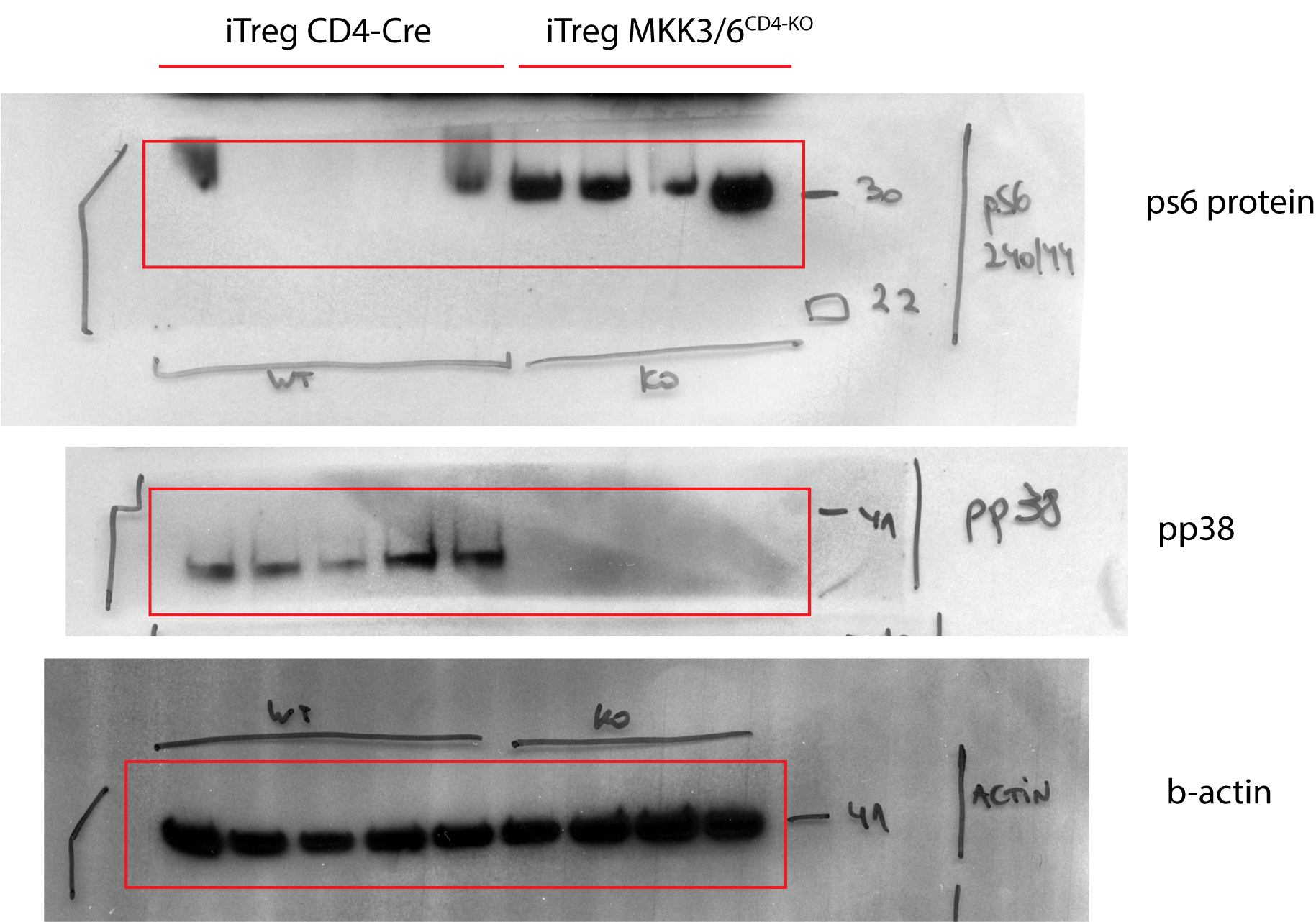

Supplement: Supplementary file 12 — Source data Fig. 7 [file 44319_2024_149_MOESM12_ESM.zip › Figure 7/7G/Unprocessed blots 7G.tif]

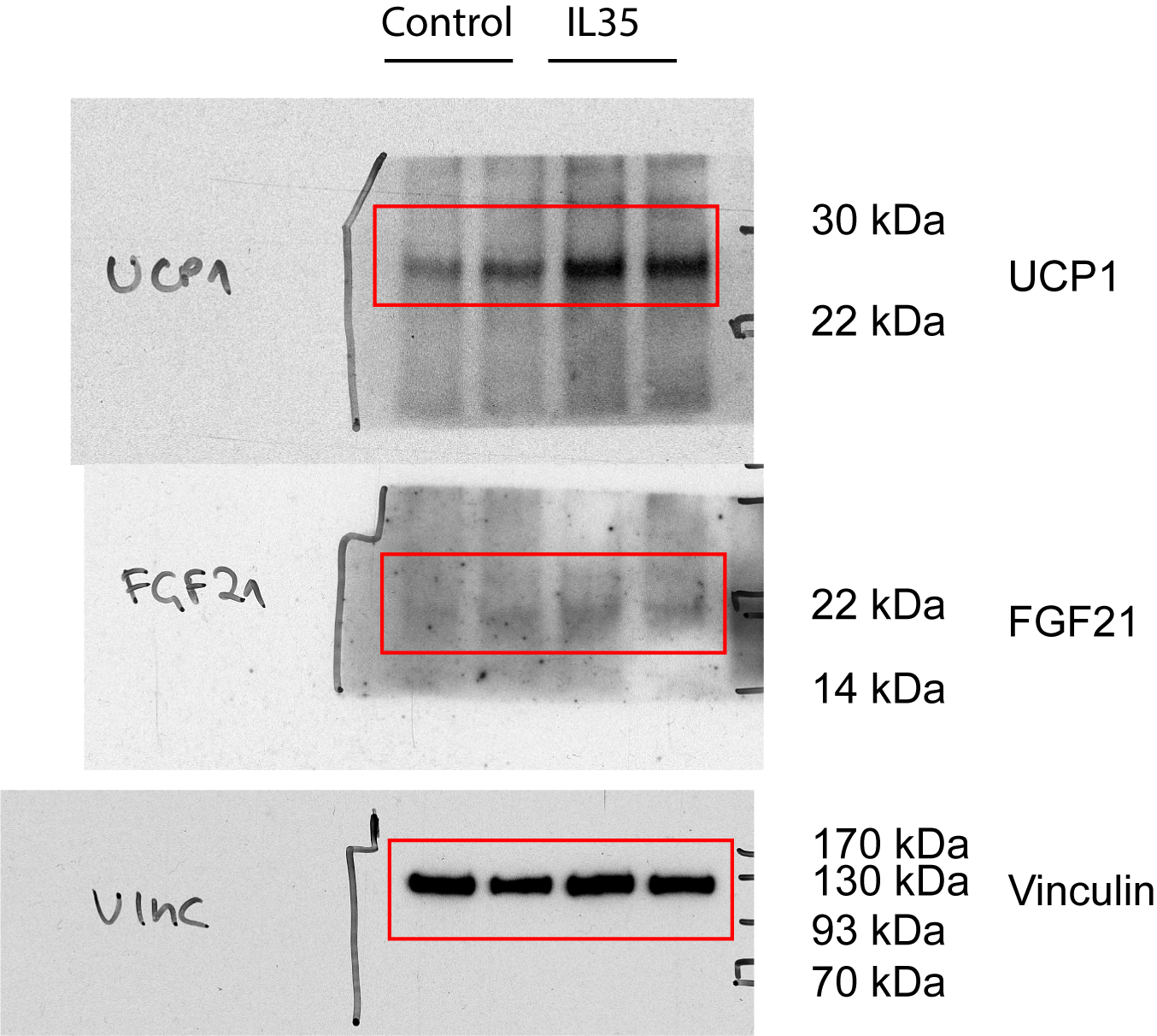

Supplement: Supplementary file 13 — Source data Fig. 8 [file 44319_2024_149_MOESM13_ESM.zip › Figure 8/8D/Unprocessed WB 8D.tif]

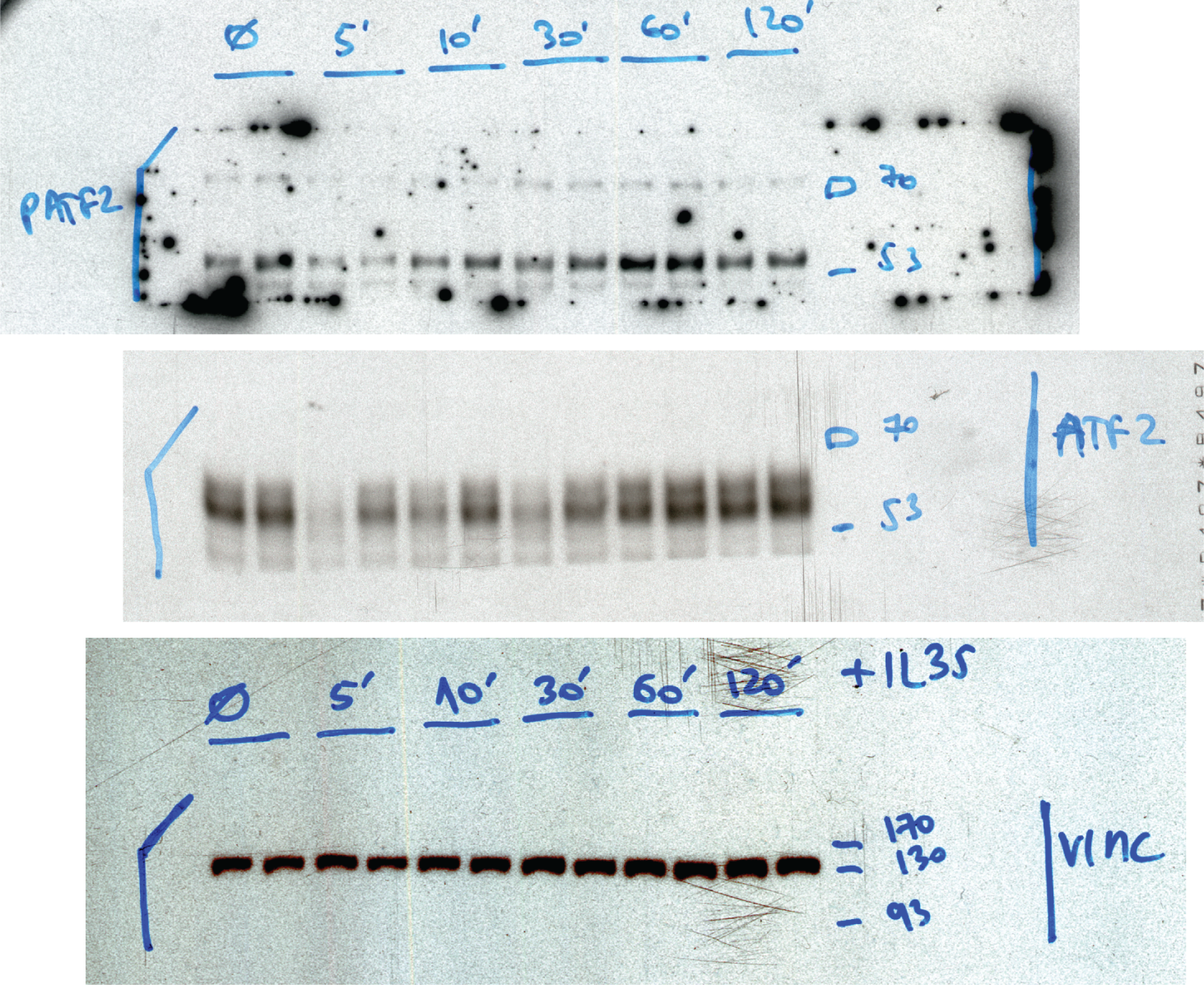

Supplement: Supplementary file 13 — Source data Fig. 8 [file 44319_2024_149_MOESM13_ESM.zip › Figure 8/8E/Unprocessed WB 8E.tif]

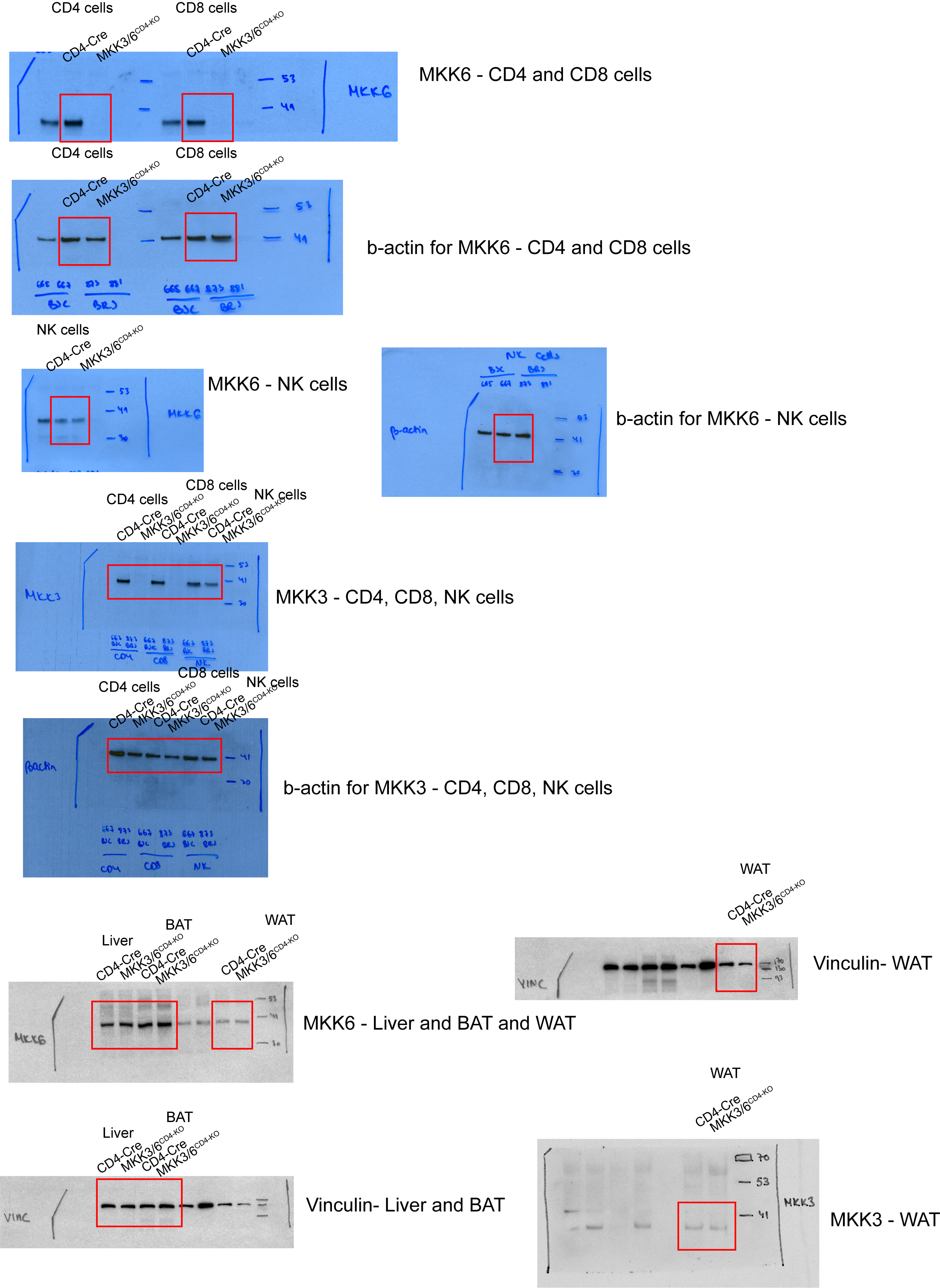

Supplement: Supplementary file 14 — Figure Source Data Appendix and EV Figures [file 44319_2024_149_MOESM14_ESM.zip › SD for Appendix and EV Figures/Appendix/Appendix Figure S1/Appendix Figure S1A/unprocessed WB S 1A.tif]

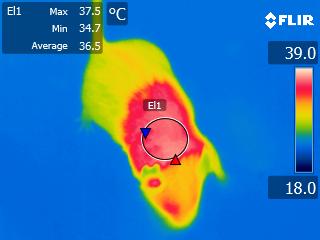

Supplement: Supplementary file 14 — Figure Source Data Appendix and EV Figures [file 44319_2024_149_MOESM14_ESM.zip › SD for Appendix and EV Figures/Expanded View Figure 1/EV 1C/CD4-Cre.jpg]

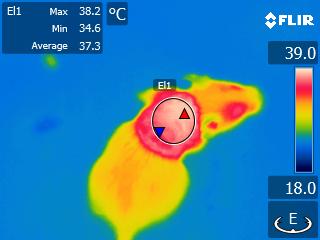

Supplement: Supplementary file 14 — Figure Source Data Appendix and EV Figures [file 44319_2024_149_MOESM14_ESM.zip › SD for Appendix and EV Figures/Expanded View Figure 1/EV 1C/MKK36CD4KO.jpg]

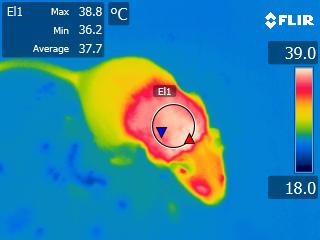

Supplement: Supplementary file 14 — Figure Source Data Appendix and EV Figures [file 44319_2024_149_MOESM14_ESM.zip › SD for Appendix and EV Figures/Expanded View Figure 1/EV 1D/MKK36CD4KO.jpg]

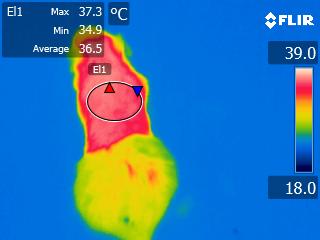

Supplement: Supplementary file 14 — Figure Source Data Appendix and EV Figures [file 44319_2024_149_MOESM14_ESM.zip › SD for Appendix and EV Figures/Expanded View Figure 1/EV 1D/MKK36f_f.jpg]

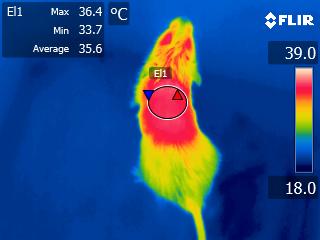

Supplement: Supplementary file 14 — Figure Source Data Appendix and EV Figures [file 44319_2024_149_MOESM14_ESM.zip › SD for Appendix and EV Figures/Expanded View Figure 2/EV 2D/CD4Cre.jpg]

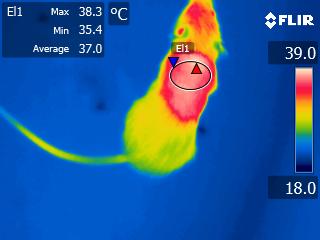

Supplement: Supplementary file 14 — Figure Source Data Appendix and EV Figures [file 44319_2024_149_MOESM14_ESM.zip › SD for Appendix and EV Figures/Expanded View Figure 2/EV 2D/MKK36CD4KO.jpg]
