# Supplementary material for: Lack of p38 activation in T cells increases IL-35 and protects against obesity by promoting thermogenesis
Source: EMBO Rep. 2024 May 10;25(6):11. doi: 10.1038/s44319-024-00149-y (PMC11169359; doi:10.1038/s44319-024-00149-y)
Supplement: Supplementary file 16 — Expanded View Figures [file 44319_2024_149_MOESM16_ESM.pdf]

Expanded View Figures

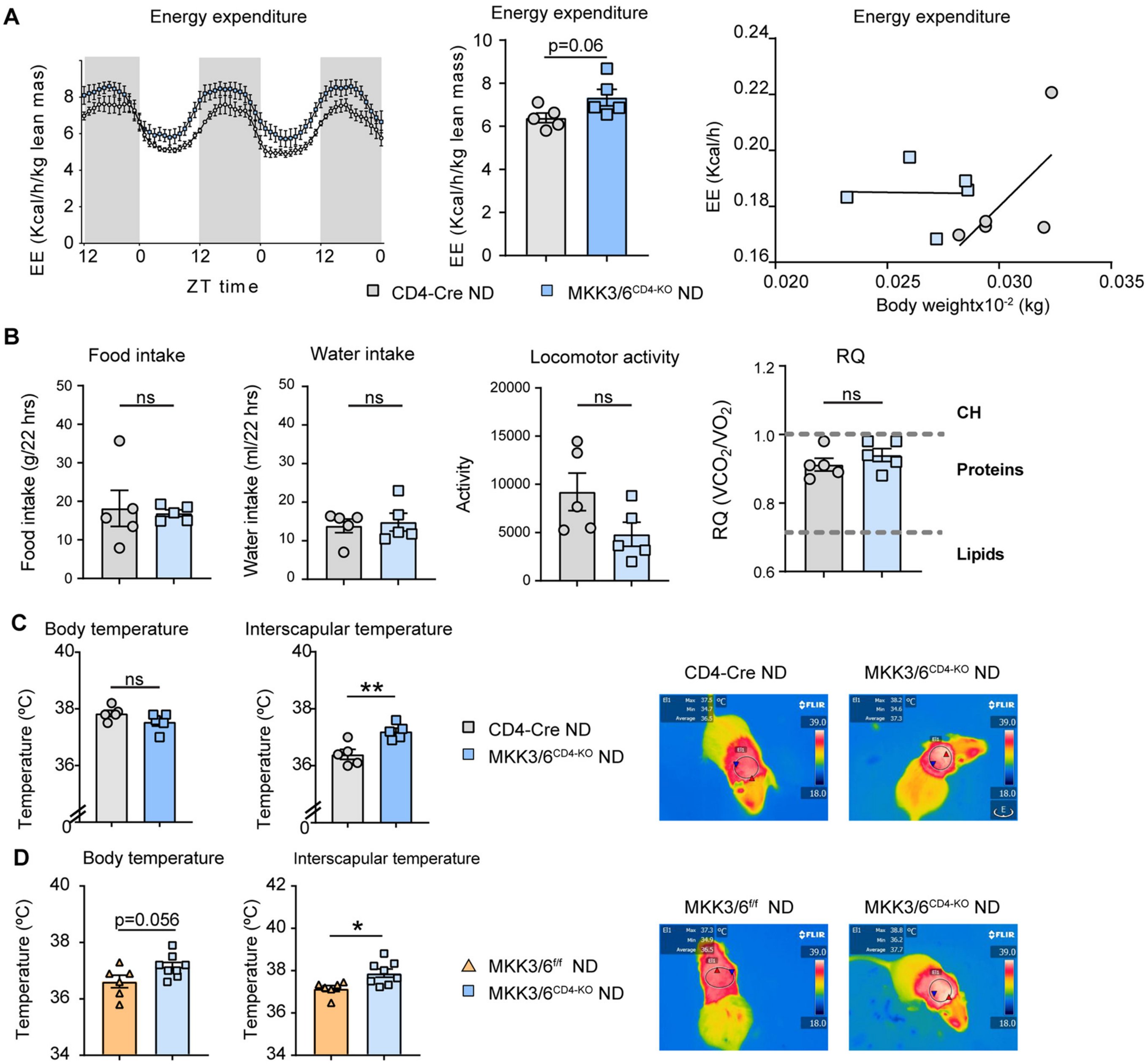

Figure EV1. MKK3/6 deficiency in T cells increases energy expenditure and BAT temperature.

(**A**) Comparison of energy balance between ND-fed MKK3/6^{CD4-KO} and CD4-Cre mice examined in a metabolic cage over a 3-day period. Hour-by-hour lean-mass-corrected variation in energy expenditure (EE) (left panel); mean lean-mass-corrected EE (middle panel); and ANCOVA analysis of EE (kcal/h) (right panel). (**B**) Food and water intake, locomotor activity, and respiratory quotient obtained from metabolic cages. (**C, D**) Body temperature and skin temperature surrounding interscapular BAT. Right panels show representative infrared thermal images in (**C**) CD4-Cre and MKK3/6 $^{CD4-KO}$ mice and (**D**) in littermates (MKK3/6$^{f/f}$) and MKK3/6^{CD4-KO} mice fed chow diet. Data information: Data are presented as mean ± SEM, *$p < 0.05$, **$p < 0.01$, ns: not significant. Exact *p*-values are shown. Analysis by 2-way ANOVA coupled to the Sidak's multiple comparison post-test (**A**) or by *t* test or by the Welch test when variances were different (**B–D**). $n = 5$–8 biologically independent mice for each group, represented as single dots in the graphs (**A–D**). Source data are available online for this figure.

 © The Author(s)

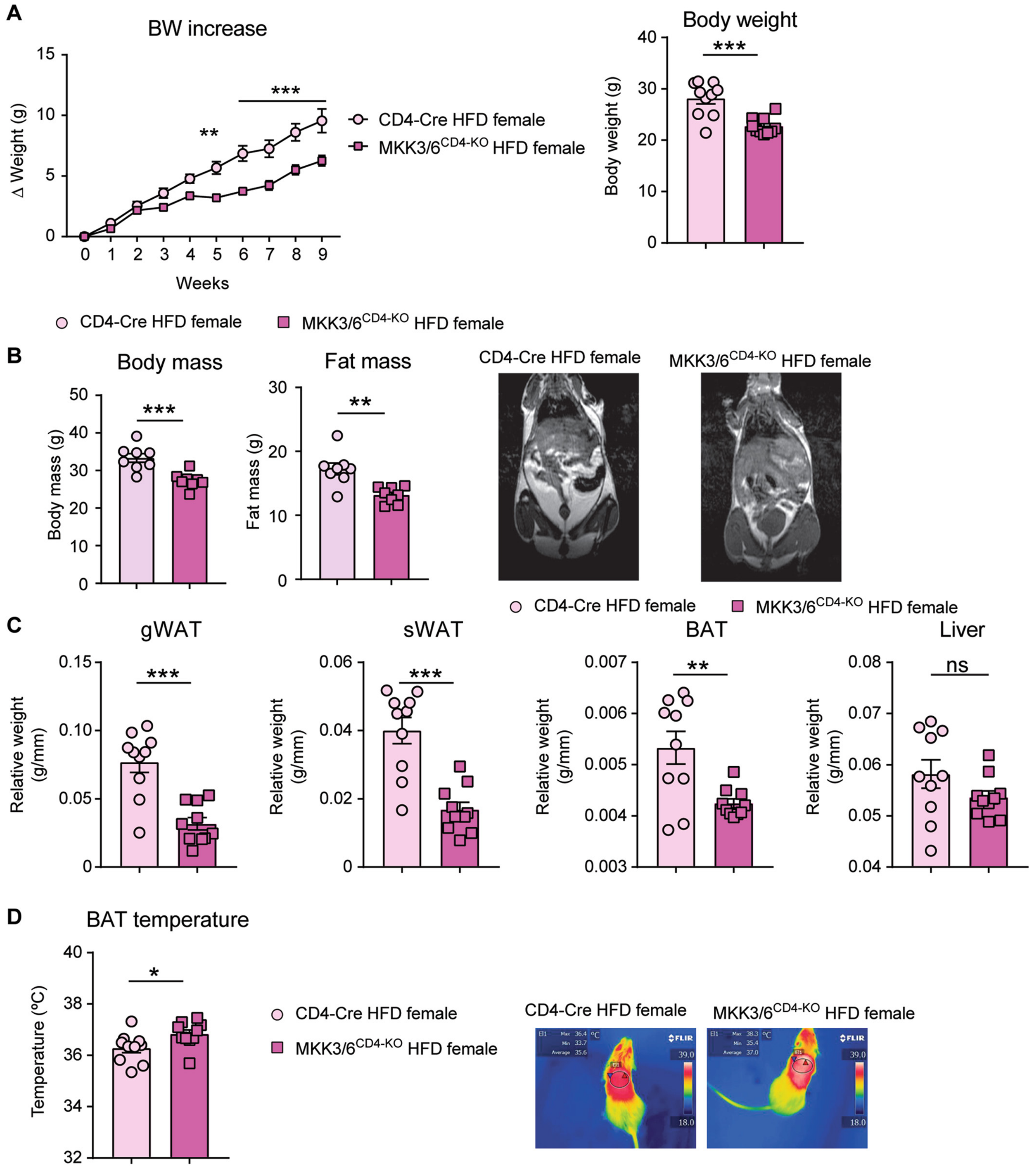

A
BW increase
Δ Weight (g)
Weeks
CD4-Cre HFD female
MKK3/6CD4-KO HFD female
Body weight
Body weight (g)
B
CD4-Cre HFD female
MKK3/6CD4-KO HFD female
Body mass
Body mass (g)
Fat mass
Fat mass (g)
C
gWAT
sWAT
BAT
Liver
Relative weight (g/mm)
ns
D
BAT temperature
Temperature (ºC)

Figure EV2. MKK3/6 deficiency in T cells protects females against HFD-induced obesity.

(**A–D**) Female MKK3/6$^{CD4\text{-}KO}$ and CD4-Cre mice were fed a high-fat diet (HFD) for 9 weeks (starting at 8–10 weeks old). (**A**) Body weight evolution in MKK3/6$^{CD4\text{-}KO}$ and CD4-Cre female mice for 9 weeks. Data are presented as the increase above initial weight (left) and absolute weight at the end of the experiment (right). (**B**) MRI analysis of body and fat mass in MKK3/6$^{CD4\text{-}KO}$ and CD4-Cre mice after 8 weeks of HFD. Representative images are shown on the right. (**C**) eWAT, sWAT, BAT, and liver mass relative to tibia length. (**D**) Skin temperature surrounding interscapular BAT. Right panels show representative infrared thermal images. Data Information: Data are presented as mean ± SEM, $^{*}p < 0.05$, $^{**}p < 0.01$, $^{***}p < 0.001$, ns: not significant. Analysis by 2-way ANOVA coupled to the Bonferroni post-test (**A**) or *t* test or by the Welch test when variances were different (**A–D**). $n = 8$–10 biologically independent mice for each group, represented as single dots in the graphs (**A–D**). Source data are available online for this figure.

 © The Author(s)

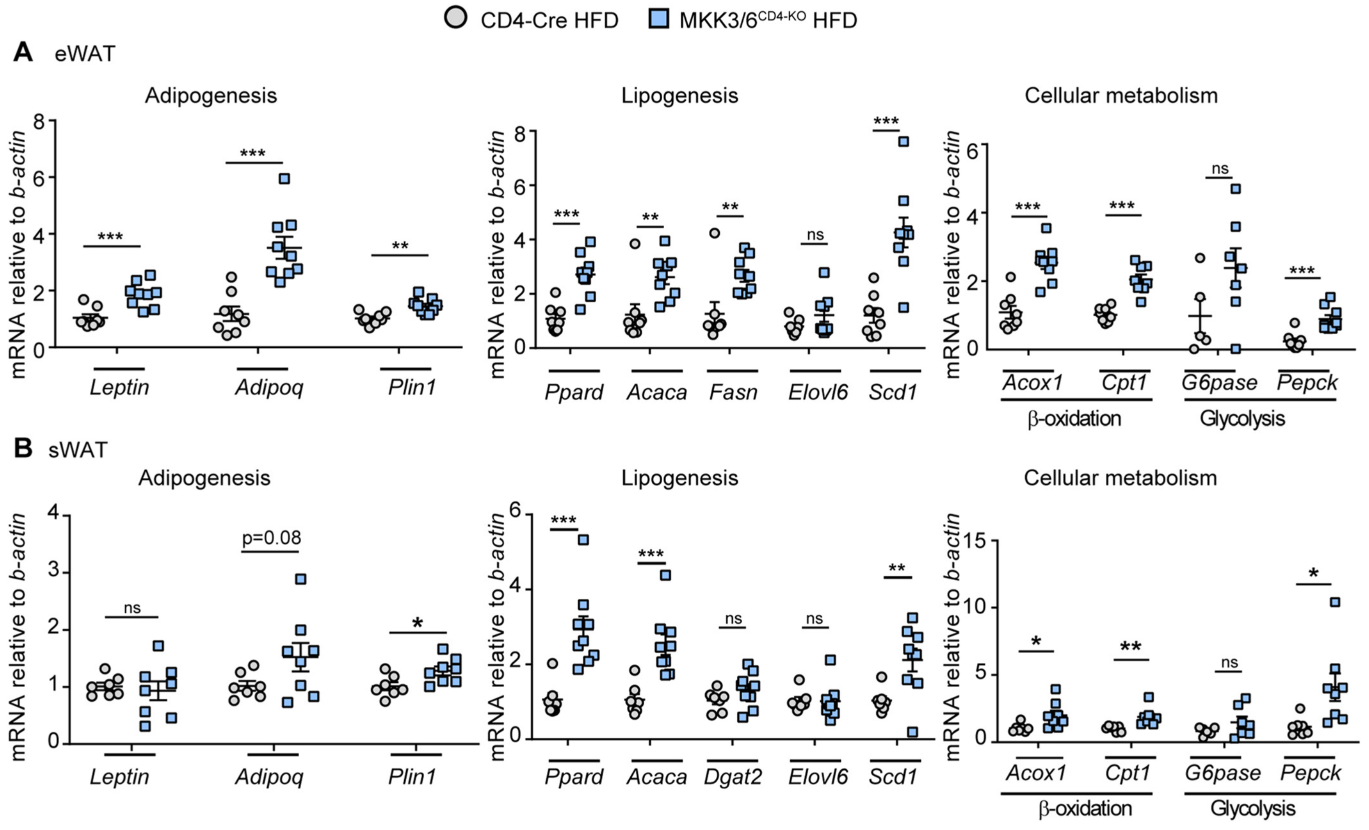

Figure EV3. Lack of MKK3/6 in T improves adipose tissue metabolic homeostasis.

(**A**, **B**) MKK3/6$^{CD4\text{-}KO}$ and control CD4-Cre mice were fed an HFD for 8 weeks. qRT-PCR analysis of adipogenic, lipogenic, β-oxidation, and glycolytic genes mRNA expression from (**A**) eWAT and (**B**) sWAT isolated from control CD4-Cre or MKK3/6$^{CD4\text{-}KO}$ mice. mRNA expression was normalized to the amount of *b-actin* mRNA. Data Information: Data are presented as mean ± SEM, $*p < 0.05$, $**p < 0.01$, $***p < 0.001$, ns: not significant. Exact *p* values are shown. Analysis by *t* test or Welch's test when variances were different. $n = 8\text{-}9$ biologically independent mice for each group, represented as single dots in the graphs. Source data are available online for this figure.

© The Author(s)

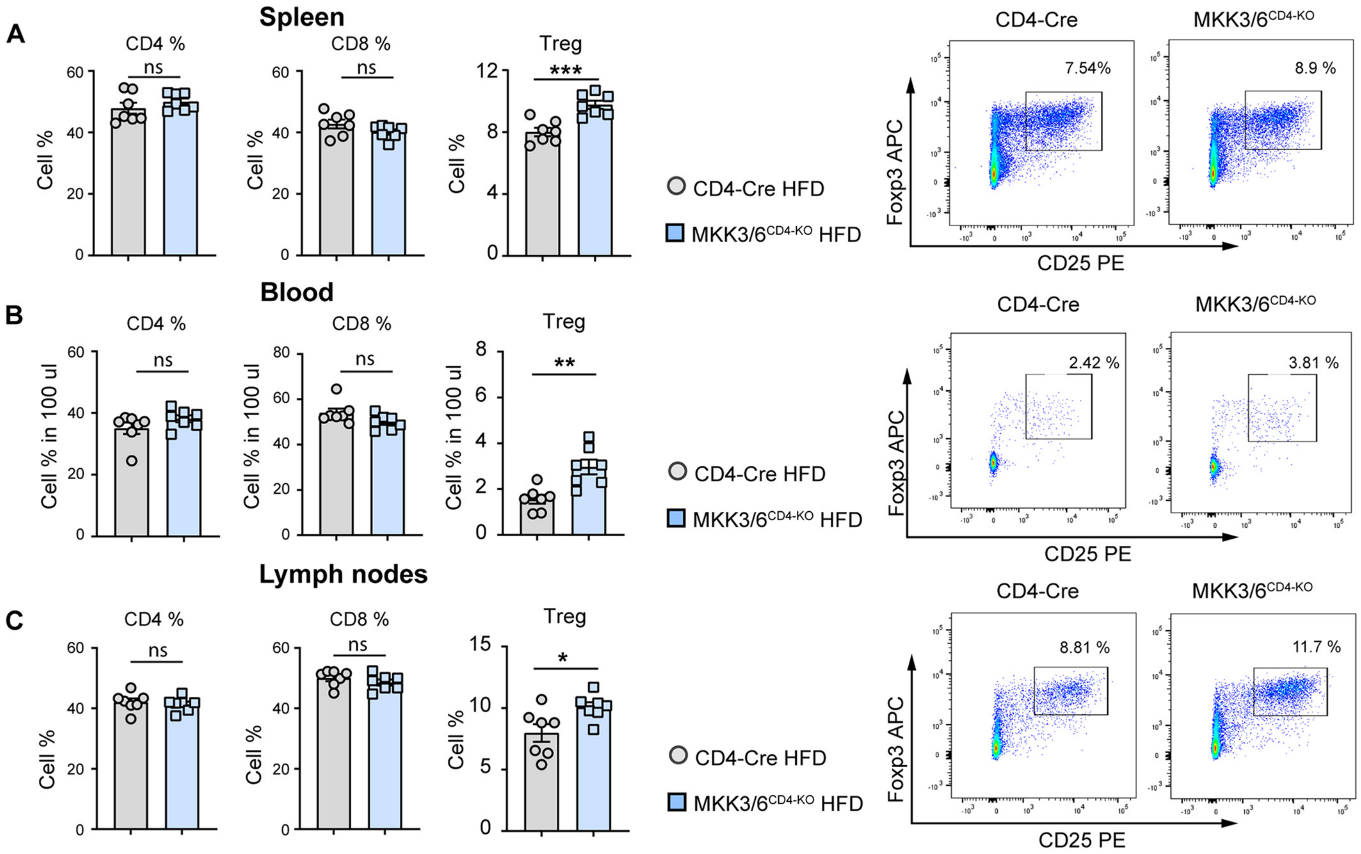

Figure EV4. MKK3/6 deletion in T cells increases Treg cell population in blood and lymph nodes.

(**A–C**) MKK3/6^{CD4-KO} and CD4-Cre mice were fed a high-fat diet (HFD) for 8 weeks. FACS quantification and representative dot plots of CD4$^+$, CD8$^+$ and Treg cells (CD4$^+$CD25$^+$Foxp3$^+$) in spleen (**A**), blood (**B**), and lymph nodes (**C**). Data Information: Data are presented as mean ± SEM, *$p < 0.05$, **$p < 0.01$, ***$p < 0.001$, ns: not significant. Analysis by *t* test or Welch's test when variances were different. $n = 7$ biologically independent mice for each group, represented as single dots in the graphs. Source data are available online for this figure.

 © The Author(s)

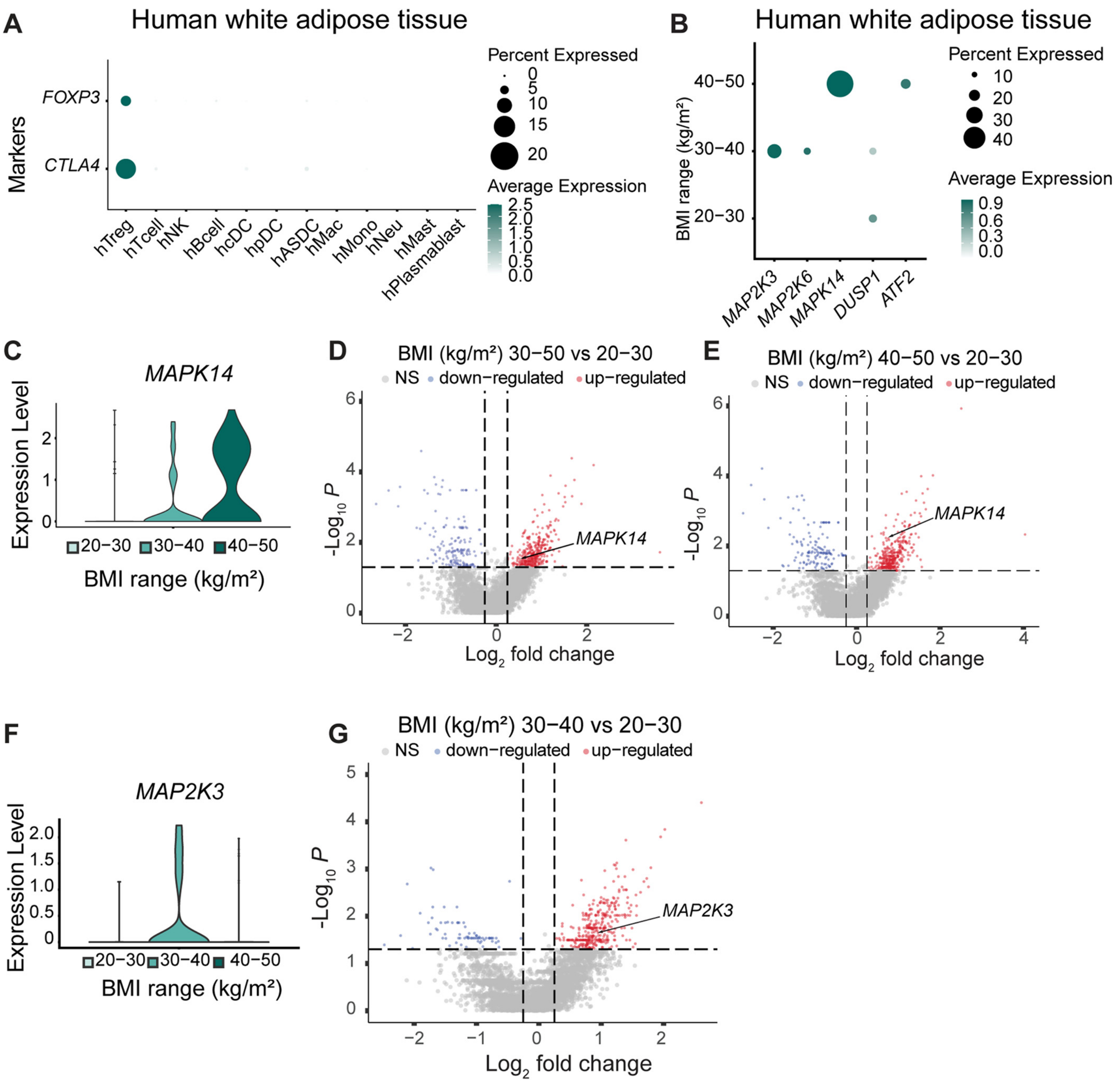

Figure EV5. p38 MAPK pathway is upregulated in Treg cells in obese human adipose tissue.

(**A–G**) The analysis was performed using human white adipose tissue single-cell RNA-seq data from Emont et al (Emont et al, 2022). (**A**) Dot plot of the expression of the indicated regulatory T cell (Treg) marker genes in the different cell type clusters. (**B**) Dot plot of the expression of the indicated genes by BMI range in human white adipose tissue Treg cluster shown in (**A**). (**C**) Violin plot showing the level of *MAPK14* gene expression by BMI range in the human white adipose tissue Treg dataset. (**D–E**) Volcano plots of differentially expressed genes in human white adipose tissue Treg subcluster in obese (BMI 30–40 kg/m^2) versus non-obese (BMI 20–30 kg/m^2) subjects (**D**) and in severe obese (BMI 40–50 kg/m^2) versus non-obese (BMI 20–30 kg/m^2) subjects (**E**). (**F**) Violin plot showing the level of *MAP2K3* gene expression by BMI range in the human white adipose tissue Treg dataset. (**G**) Volcano plots of differentially expressed genes in human white adipose tissue Treg subcluster in class 1 and 2 obesity (BMI 30–40 kg/m^2) versus non-obese (BMI 20–30 kg/m^2) subjects. The vertical dashed lines in (**D**, **E**, **G**) indicate a log2 fold change cut-off of 0.25. The horizontal dashed lines in (**D**, **E**, **G**) indicate a −log10 *p*-value cut-off of 1.3 (*p*-value < 0.05). Data information: Differentially expressed genes between BMI ranges were identified with a non-parametric Wilcoxon rank sum test. Obese (BMI 30–40 kg/m^2) $N = 3$ biologically independent patients; Severe obese (BMI 40–50 kg/m^2) $N = 6$ biologically independent patients; non-obese (BMI 20–30 kg/m^2) $N = 5$ biologically independent patients. DC: dendritic cells; Mac: macrophages; Mono: monocytes; Neu: neutrophiles; Mast: mastocytes.
